# Purine and Pyrrolopyrimidine‐Based Small Molecules as Multitarget Therapeutics

**DOI:** 10.1002/ardp.70296

**Published:** 2026-07-09

**Authors:** Federica Borghi, Antonio Laus, Claudia Sorbi, Giulio Rastelli

**Affiliations:** ^1^ Department of Life Science (DSV) University of Modena and Reggio Emilia Modena Italy; ^2^ Center for Advanced Studies Research and Development in Sardinia (CRS4) Pula Italy

**Keywords:** drug design, polypharmacology, purines, pyrrolopyrimidines, structure–activity relationships

## Abstract

Purines and pyrrolo[2,3‐*d*]pyrimidines are privileged heterocyclic scaffolds widely represented in small molecules targeting a broad range of therapeutically relevant targets, including kinases, folate‐metabolizing enzymes, histone deacetylases (HDACs), bromodomain containing proteins, membrane transporters, phosphodiesterases (PDEs), ion channels, and toll‐like receptors (TLRs). While these scaffolds have been extensively investigated in medicinal chemistry, their role in multitarget drug design has received comparatively less attention. This review provides a comprehensive overview of purine‐ and pyrrolopyrimidine‐based compounds reported over the last two decades from a multitarget medicinal chemistry perspective, with particular emphasis on the design principles and SAR underlying simultaneous modulation of multiple biological targets. By systematically comparing compounds across different target classes, we highlight the key structural features responsible for multitarget activity and discuss how specific scaffold modifications influence potency, selectivity, and polypharmacological profiles. Representative examples are presented to illustrate the successful application of these scaffolds in the development of dual‐ and multitarget inhibitors with promising anticancer, anti‐inflammatory, and drug resistance‐overcoming activities. Particular attention is devoted to compounds capable of overcoming drug resistance through the simultaneous modulation of complementary biological pathways. Overall, this review provides an integrated medicinal chemistry framework for understanding multitarget purine‐ and pyrrolopyrimidine‐based ligands and identifies emerging opportunities and challenges for the development of next‐generation polypharmacological agents.

## Graphical abstract

Purine and pyrrolopyrimidine scaffolds target multiple oncogenic pathways: cyan kinases (VEGFR‐2, Aurora A, JAK2); red epigenetic/chaperone regulators (HDAC6, BRD4, Hsp90); blue efflux and signaling/sensory modulators (MRP1, PDE, TLR, TRPA1); yellow folate‐pathway enzymes (TS, DHFR, GARFTase), defining a dual‐scaffold platform for multi‐target anticancer therapy.

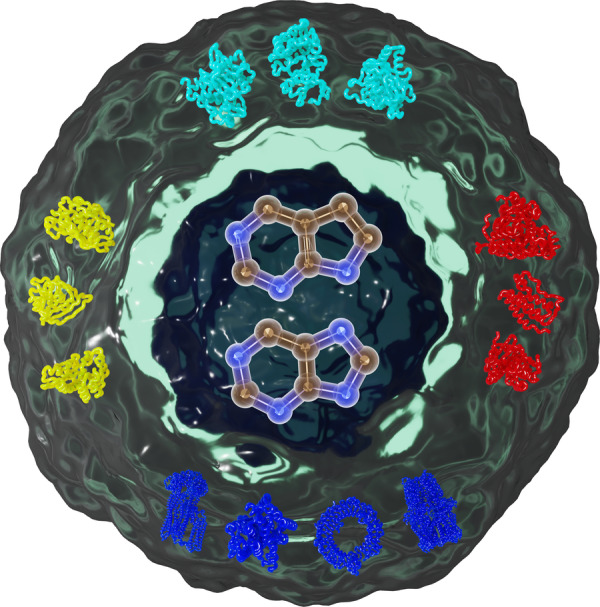

## 1 Introduction

Purines and pyrrolo[2,3‐*d*]pyrimidines (pyrrolopyrimidines) represent two privileged heterocyclic scaffolds that play significant roles in drug design and discovery. Purines, exemplified by adenine and guanine, are fundamental components of nucleic acids and play key roles in cell signaling and numerous physiological processes [1]. In parallel, pyrrolopyrimidines have emerged as versatile compounds with broad pharmacological profiles, including anticancer, antiviral, antibacterial, and anti‐inflammatory activities [2]. Owing to their structural similarity to endogenous purines and their capacity to engage nucleotide‐recognizing proteins, both scaffolds have attracted considerable interest in the design of multi‐target ligands.

This review systematically examines the binding properties and multi‐target potential of pyrrolopyrimidine and purine derivatives across a wide range of therapeutically relevant targets. These targets include protein kinases, folate enzymes, regulators of gene expression and epigenetics (e.g., HDACs and BRD), membrane transport proteins (e.g., ABC transporters), regulators of intracellular second messenger levels (e.g., PDE), ion channels, sensory receptors (e.g., TRPA1), and receptors involved in immune and inflammatory response (e.g., TLR). The selected targets were chosen based on their relevance to current therapeutic challenges, documented evidence of multi‐target activity, and translational potential. Particular emphasis is placed on studies reported over the past two decades.

These protein classes regulate essential cellular processes. Protein kinases [3, 4] and folate‐metabolizing enzymes [5, 6, 7] play a crucial role in controlling cell proliferation and nucleotide biosynthesis. Epigenetic regulators [8, 9], including histone‐modifying enzymes [10, 11], modulate gene transcription and cellular differentiation, providing opportunities for therapeutic intervention in cancer and inflammatory disorders. Membrane transport proteins [12] and cyclic nucleotide–modulating enzymes regulate intracellular signaling and drug distribution [13], while ion channels and sensory receptors control ion fluxes and signal transmission [14]. In addition, immune and inflammatory receptors regulate host defense mechanisms and inflammatory responses [15]. Dysfunctions in these interconnected systems contribute to disease development and progression, highlighting their relevance for multi‐target therapeutic strategies [3, 4, 5, 6, 7, 8, 9, 12, 13, 14, 15].

The chemical exploration and optimization of purine and pyrrolopyrimidine‐based multi‐target agents therefore represent a promising strategy for the development of next‐generation therapeutics with improved efficacy and reduced toxicity [16, 17]. This review highlights how structural modifications within these scaffolds enable engagement of distinct yet mechanistically linked biological targets [18]. By consolidating current advancements in multi‐target purine‐ and pyrrolopyrimidine‐based compounds, we aim to provide medicinal chemistry insights that may guide the design and development of more effective therapeutic agents for complex diseases, where single‐target approaches have shown limited success.

## 2 Pyrrolopyrimidines

### 2.1 As Multi‐Kinase Inhibitors

Kinases are one of the largest and most functionally diverse gene families, essential for transmitting signals and regulating complex cellular processes. They are critical in metabolism and in many other cellular pathways involved in cell growth and death. Therefore, targeting kinases is a well‐established therapeutic strategy, mostly in cancer treatment, and their defects may cause various diseases. The pyrrolo[2,3‐d]pyrimidine core belongs to the privileged class of ATP‐competitive scaffolds and has been widely explored for its ability to engage multiple kinases, including receptor tyrosine kinases (VEGFR‐2, EGFR, PDGFRβ, VEGFR‐1) and non‐receptor kinases (CDK2, aurora kinases A/B). By exploiting conserved hydrogen‐bond networks (hinge region, DFG motif) while taking advantage of additional hydrophobic pockets, unique to each kinase, small modifications at positions 2, 4, or 6 of the pyrrolo[2,3‐d]pyrimidine ring proved able to modulate selectivity and potency against kinase targets.

**Figure 1:**
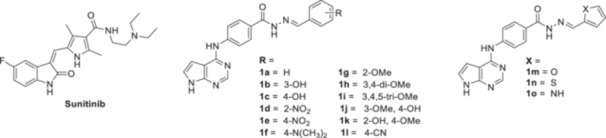
Sunitinib and general structure of pyrrolopyrimidine derivatives as multi‐kinase inhibitors.

In 2023, *Alotaibi* et al. reported the discovery of new anticancer agents by modifying sunitinib's chemical structure, a potent and well‐known multitarget tyrosine kinase inhibitor [19, 20]. A set of 15 new pyrrolo[2,3‐d]pyrimidine‐based derivatives was developed as multi‐kinase inhibitors (Figure 1). Compounds **1f**, **1l**, and **1n** effectively targeted EGFR (epidermal growth factor receptor, IC_50_ = 0.15 µM for **1f**, 0.07 µM for **1l**, 0.03 µM for **1n**), HER2 (human epidermal growth factor receptor 2, IC_50_ = 0.04 µM for **1f**, 0.04 µM for **1l**, 0.03 µM for **1n**), VEGFR‐2 (Vascular endothelial growth factor receptor 2, IC_50_ = 0.03 µM for **1f**, 0.09 µM for **1l**, 0.07 µM for **1n**), and CDK2 (cyclin‐dependent kinase 2, IC_50_ = 0.40 µM for **1f**, 0.43 µM for **1l**, 0.13 µM for **1n**) similarly to sunitinib, while **1j** and **1c** showed selective VEGFR‐2 (IC_50_ = 0.05 µM for **1j**, 0.307 µM for **1c**) inhibition, and **1i** inhibited both HER2 and VEGFR‐2 (IC_50_ = 0.41 µM and 0.19 µM respectively).

**Figure 2:**
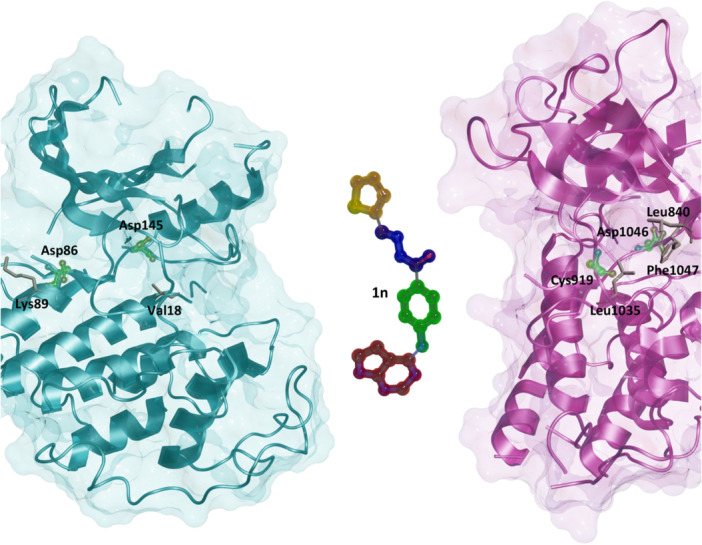
Structural representation of compound **1n**, rationally designed to simultaneously target both CDK2 and VEGFR‐2. The carbon atoms in its substructures are distinctly colored according to their corresponding interaction regions: hinge region (red), linker (green), DFG domain (blue), and allosteric binding region (yellow). The proteins are shown in cartoon style: CDK2 in cyan and VEGFR‐2 in pink. Amino acid residues involved in hydrogen‐bond interactions are depicted in ball‐and‐stick style with carbon atoms shown in green, whereas residues mediating hydrophobic interactions are represented entirely in gray.

Docking studies with CDK2 showed that compound **1f** binds similarly to sunitinib, forming hydrogen bonds with Leu83 and Asp145. Compound **1n** (Figure 2), on the other hand, interacts with CDK2 through Asp145 and Asp86 and also engages residues such as Val18 and Lys89, suggesting a different binding mode. For VEGFR‐2, compounds **1c** and **1j** bind through water‐mediated hydrogen bonds to Asp1045, Glu885, and Cys919. Interestingly, **1n** showed fewer hydrogen bonds yet maintained strong inhibitory activity, indicating that small structural differences (e.g., para‐hydroxyl group *vs* tertiary amine) can significantly impact kinase recognition and activity.

The synthesized compounds exhibited various cytotoxic effects on different cancer cell lines, including breast cancer (MCF‐7 and MDA‐MB‐231), hepatocellular carcinoma (HepG2), and epithelioid cervical carcinoma (HeLa). **1c**, **1f**, **1j**, and **1n** demonstrated strong antiproliferative activitiy, similar to sunitinib, in particular, **1f** and **1n** halted cell‐cycle progression (G2/M‐phase) and induced apoptosis in HepG2 cells by increasing pro‐apoptotic proteins (Caspase 3, Bax) and decreasing the anti‐apoptotic protein Bcl‐2. These effects are consistent with their combined inhibition of CDK2 and VEGFR‐2.

**Figure 3:**
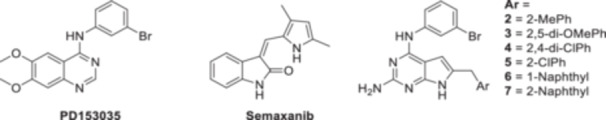
PD153035, semaxanib and general structures of RTKs inhibitors by Gangjee et al. [21, 22].

In 2003, *Gangjee* et al. developed a unified model to understand how angiogenesis‐related tyrosine kinase receptors (RTKs) interact with ATP. They subsequently synthesized several compounds (Figure 3) to inhibit multiple RTKs simultaneously, with the aim to overcome drug resistance and improve therapeutic safety [21, 22, 23]. These compounds were tested on human cancer cell lines that express high levels of RTKs. Compounds **2** and **3** displayed comparable inhibitory activity against VEGFR‐2 (IC_50_ = 0.25 µM and 0.62 µM, respectively), showing better activity compared with reference compounds PD153035 and semaxanib [24, 25]. Compound PD153035 is an EGFR inhibitor featuring a quinazoline core with substituents that enhance its affinity for EGFR. In contrast, semaxanib is a VEGFR‐2 inhibitor with an indolyl‐quinazoline structure, which better matches the VEGFR‐2 binding characteristics. Compounds **2–7** are pyrrolopyrimidines that structurally differ from PD153035 and semaxanib. This core modification significantly influenced kinase selectivity and potency. Compounds **2** and **3** contain substituents at the 2‐position of the pyrrolopyrimidine ring, particularly aryl or substituted aryl groups, which enhance interactions with VEGFR‐2. Compound **3** showed superior potency over both PD153035 and semaxanib, suggesting that its specific C‐2 substitution optimizes VEGFR‐2 activity. Computational modeling suggested that this improved potency comes from an extra hydrogen bond formed by the 2‐NH_2_ group with the hinge region. Compound **4** demonstrated EGFR inhibition (IC_50_ = 0.23 µM while 28.11 µM for VEGFR‐2), indicating that its substitution pattern preserves key pharmacophoric elements for EGFR binding, similar to PD153035. Compounds **5** and **6** are dual inhibitors of VEGFR‐1 (Flt) (IC_50_ = 26.8 µM for 5 and 19.2 µM for 6) and VEGFR‐2 (IC_50_ = 5.58 µM for 5 and 5.08 µM for 6), suggesting that their C‐2 and/or C‐4 substitutions allow simultaneous binding to both kinases. The presence of specific alkyl, aryl, or heterocyclic groups may enable these compounds to accommodate structural differences between VEGFR‐1 and VEGFR‐2 binding sites. Compounds **2** and **4** were particularly effective against A431 cells (human epidermoid carcinoma) overexpressing EGFR, thus surpassing conventional RTK inhibitors. Compound **7** exhibits a dual‐targeting effect on EGFR and VEGFR‐1 (IC_50_ = 1.24 µM and 15.2 µM, respectively). A key C‐2 modification likely enables this multi‐target activity by enhancing fit with both EGFR and VEGFR‐1 binding pockets [21, 22, 23]. Compounds **6** and **7** demonstrated dual‐targeting capability by inhibiting VEGFR‐1, illustrating how minor shifts in substituents can modulate kinase selectivity.

**Figure 4:**
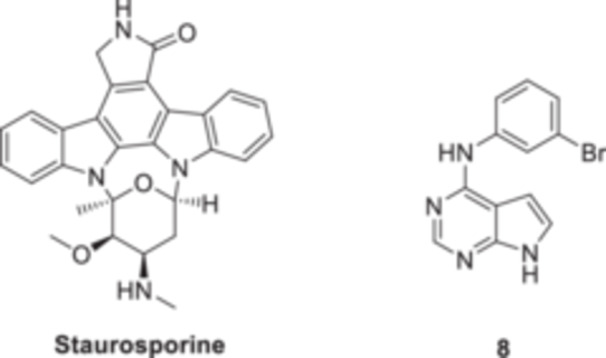
Staurosporine and structure of **8** as EGFR and AURKA inhibitor.

In 2018, *Kurup* et al. discovered additional kinase inhibitors containing a pyrrolo[2,3‐*d*]pyrimidine scaffold to achieve dual inhibition of EGFR and aurora kinase A (AURKA) [26]. Compared with the reference staurosporine [27], these compounds showed EGFR and AURKA inhibition at nanomolar and micromolar levels, respectively. Compound **8** was identified as the most active dual inhibitor in the series, exhibiting moderate AURKA inhibition (IC_50_ = 2 µM) alongside strong EGFR inhibition (IC_50_ = 3.8 nM; Figure 4). Molecular modeling studies indicated critical interactions with hinge residues Met793 in EGFR and Ala213 in AURKA. Its sub‐10 nM EGFR inhibitory activity is associated with binding to the hydrophobic cleft, similarly to known EGFR inhibitors, while its weaker potency against AURKA is likely due to fewer hydrogen‐bond interactions. It was further evaluated in four squamous cell head and neck cancer cell lines (SCCHN) to study the downstream effects of the EGFR and AURKA inhibition. Compound **8** inhibited EGFR and AURKA mediated phosphorylation events in SCCHN cells at 100 µM concentration, suggesting limited cellular activity [26]. The authors did not report a broader kinase‐selectivity profile and the compound's limited substitution (i.e., relatively few side‐chain modifications) implies the need for further functionalization to improve cellular potency and selectivity.

The ability of the pyrrolopyrimidine‐based inhibitors described in this section to induce apoptosis and inhibit cell proliferation by effectively targeting multiple protein kinases highlights their overall potential in overcoming drug resistance and enhancing treatment specificity. Through precise adjustments in ring substitutions, such as 2‐amino, 4‐anilino, or 6‐benzyl groups, researchers have successfully fine‐tuned kinase selectivity and potency across the series, as exemplified by the effectiveness of compounds **1f**, **1n**, **3**, **4**, and **8**.

**Figure 5:**
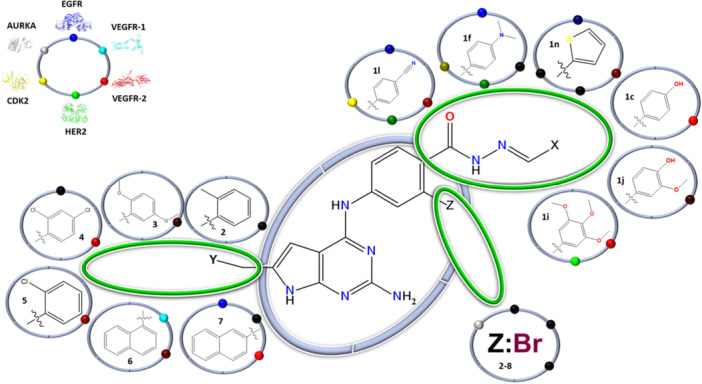
First map of SAR for the pyrrolopyrimidine chemotype. The central panel shows the 2D core scaffold; green ovals highlight substituents that are required for binding and inhibitory activity of membrane receptor tyrosine kinases (RTKs) and intracellular Ser/Thr kinases. Peripheral gray circles show the representative substituents explored with different substitution sites. For each substituent, colored dots indicate the kinase evaluated (EGFR, blue; HER2, green; VEGFR‐1, cyan; VEGFR‐2, red; CDK2, yellow; AURKA, gray) that has a fixed position on the gray circle. The compound's potency is encoded by darkening‐to‐black each colored dot (darker = higher potency), enabling rapid identification of multitarget profiles and relative strength against each kinase. Color assignment and position for each kinase are depicted apart in the gray circle at the top left of the figure.

A visual overview of the structure–activity relationship (SAR) analysis for the investigated chemotypes **1**–**8** is provided in Figure 5.

**Figure 6:**
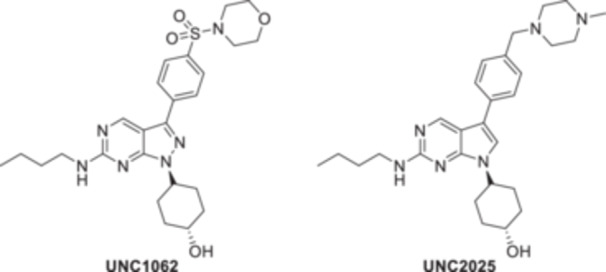
Structures of UNC1062 and UNC2025 inhibitors.

The tyrosine kinase receptors include members of the TAM family (TYRO3, AXL and MER), FLT3, and many others. Dysregulated expression and activation of MER have been linked to the development of several cancers, including acute lymphoblastic leukemia (ALL), acute myeloid leukemia (AML), non‐small cell lung cancer (NSCLC), melanoma, and glioblastoma. In these malignancies, MER plays a key role in enhancing cancer cell survival, contributing to tumor progression and resistance to chemotherapy [28]. Likewise, activating mutations in FLT3, particularly internal tandem duplications (ITD) in the juxtamembrane domain, are found in about 30% of adult and 15% of pediatric AML cases. In AML, FLT3‐ITD (Fms‐Like Tyrosine kinase 3—Internal Tandem Duplication) is recognized as a key oncogenic driver. Thus, dual inhibition of these targets may enhance therapeutic efficacy, particularly in AML, by simultaneously blocking multiple oncogenic pathways [28]. In 2014, *Zhang* et al. developed UNC2025, a potent and orally bioavailable MER/FLT3 dual inhibitor (MER IC_50_ = 0.74 nM and FLT3 IC_50_ = 0.80 nM) [28]. This successful lead was the result of sequential SAR optimization starting from the potent UNC1062 [29], a pyrazolopyrimidine derivative (Figure 6). While UNC1062 was highly active against both MER (IC_50_ = 1.1 nM) and FLT3 (IC_50_ = 3.0 nM), its poor solubility and negligible oral exposure necessitated chemical modifications, which led to the discovery of UNC2025 with improved drug metabolism and pharmacokinetic parameters. Kinome profiling against over 300 kinases revealed that MER and FLT3 were the most potently inhibited targets, indicating a dual‐inhibitory profile, which was subsequently confirmed by targeted IC_50_ measurements and cell‐based assays. Moreover, after oral administration, UNC2025 effectively inhibited MER phosphorylation as confirmed by pharmacodynamic studies assessing phospho‐MER levels in leukemic blasts isolated from mouse bone marrow. The authors also established a cost‐effective synthetic route to obtain a suitable quantity of UNC2025 for in vivo preclinical studies [28].

HCK (hematopoietic cell kinase), together with FLT3‐ITD, represents a critical oncogenic target in several hematological malignancies. In 2017, *Koda* et al. synthesized new derivatives of 7‐cyclohexyl‐5‐(4‐phenoxyphenyl)‐7H‐pyrrolo[2,3‐d]pyrimidine‐4‐amine by investigating 15 different amino acids linked to the cyclohexyl moiety to modulate both physicochemical properties and binding interactions with HCK (Table 1) [30]. The rationale for introducing amino acids was to increase hydrophilicity, optimize hydrogen‐bonding interactions with key residues such as Asp348, and to balance lipophilicity to improve cellular uptake. Molecular docking studies on the previously reported HCK inhibitor RK‐20449 revealed that its highly potent inhibitory activity (IC_50_ = 0.43 nM) is mostly due to a piperazine moiety interacting with the Asp348 residue of HCK [31]. In an attempt to optimize this molecule, the authors introduced structural variations at position 4 of the cyclohexyl ring to increase hydrophilicity. These chemical modifications included different amino acids such as alanine, phenylalanine, proline, and tyrosine.

**Table 1:** Structure and biological activity of **9‐14** and RK 20449 as potent HCK and FLT3‐ITD dual inhibitors.

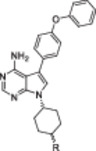					
Compound	R			IC_50_ (nM)	
				HCK	FLT3‐ITD
9	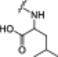	*L*‐Leu	*trans*	4.4	14
10	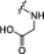	Gly	*trans*	3.7	28
11	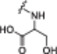	*L*‐Ser	*trans*	1.8	18
12	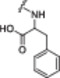	*L*‐Phe	*trans*	7.5	24
13	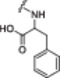	*D*‐Phe	*trans*	18	2.6
14	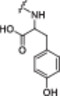	*D*‐Tyr	*cis*	13	5
RK‐20449	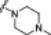	—	*trans*	0.43	24

Furthermore, it was observed that replacing the original piperazine of RK‐20449 with acyclic amines, HCK potency decreased but a modest FLT3‐ITD inhibition was maintained. The presence of a carboxylic acid group in the aminoalkyl chain, as in compound **9**, improved HCK activity through hydrogen bonding with residues such as Asp348, but the overall inhibitory potency remained slightly lower than compound RK‐20449. Lastly, retaining the phenoxyphenyl group at position 5 of the pyrrolo[2,3‐d]pyrimidine scaffold maintained potency against FLT3‐ITD. The stereochemistry of the cyclohexyl ring strongly influenced HCK inhibition, with *trans* isomers generally outperforming *cis* ones for optimal hydrogen‐bonding interactions with Asp348. Interestingly, FLT3‐ITD potency is less dependent on stereochemistry. Compounds **9** and **14** showed critical hydrogen bonding between the amino substituent and Asp348, particularly in *trans* isomers. Notably, the amino and the carboxyl groups of tyrosine in compound **14** interact with the carbonyl of Ala390 *via* a water molecule, and the phenol hydroxyl group of **14** interacts with the carbonyl of Gly279 in the HCK active site, resulting in a significant HCK inhibition. Aromatic amino acids such as phenylalanine or tyrosine (compounds **12**–**14**) further boosted FLT3‐ITD activity through π‐interactions. Compounds **9–12** displayed more potent HCK inhibition compared with compound **14**. Compounds **9** and **14** exhibited strong cytotoxicity against MV4‐11 cells (human acute monocytic leukemia), in agreement with their potent dual inhibition of HCK and FLT3‐ITD. In contrast, compounds with weaker cellular potency, such as **10** and **11**, underlined the importance of balancing enzyme inhibition with appropriate drug‐like properties, including cell permeability and lipophilicity. In conclusion, the SAR analyses led to the identification of compound **14** endowed with potent dual HCK and FLT3‐ITD inhibitory activity and strong MV4‐11 cytotoxicity [2, 30]. Therefore, the pyrrolo[2,3‐d]pyrimidine scaffold is highly adaptable to achieve dual inhibition of HCK and FLT3‐ITD, offering promising results for treating hematological malignancies. The careful optimization of substituents and stereochemistry may enhance the potency and improve cellular activity.

**Figure 7:**
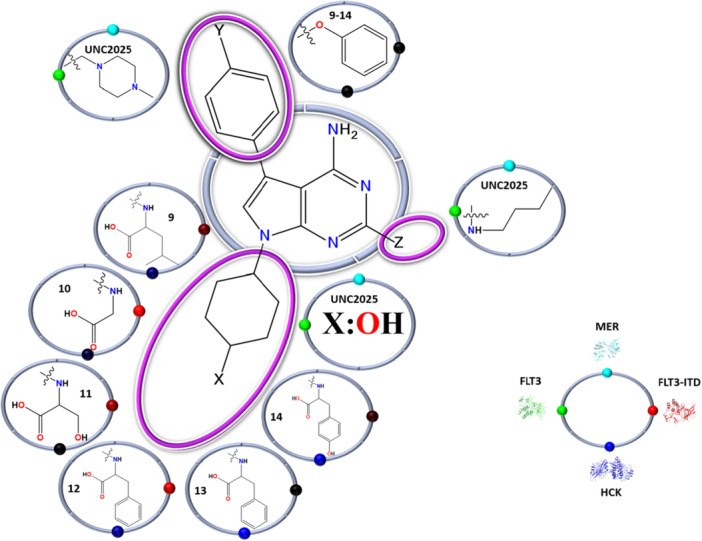
Second map of SAR for the pyrrolopyrimidine chemotype. The central panel shows the 2D core scaffold; purple ovals denote structural determinants required for binding and inhibitory activity of hematopoietic/immune tyrosine kinases. Peripheral gray circles show the representative substituents explored with different substitution sites. For each substituent, colored dots indicate the kinase evaluated (MERTK, cyan; FLT3, green; FLT3‐ITD, red; HCK, blue) that has a fixed position on the gray circle. The compound's potency is encoded by darkening‐to‐black each colored dot (darker = higher potency), enabling rapid identification of multitarget profiles and relative strength against each kinase. Color assignment and position for each kinase are depicted apart in the gray circle at the bottom right of the figure.

A visual overview of the SAR analysis for the investigated chemotypes **9**–**14** and UNC2025 is provided in Figure 7.

### 2.2 As Antifolates

The potency of pyrrolopyrimidine‐based antifolates arises from hydrogen‐bonding interactions involving key ring substituents such as the 2‐amino group, the 4‐oxo moiety, and the ring nitrogens N1, N3, N7, which contribute differently depending on the enzyme. All these recognize folate‐dependent enzymes, thymidylate synthase (TS), dihydrofolate reductase (DHFR), and de novo purine biosynthetic enzymes such as GARFTase and AICARFTase. Subtle modifications to the core structure (e.g., switching from pyrrolo[3,2‐*d*]pyrimidine to pyrrolo[2,3‐*d*]pyrimidine), or introduction of small alkyl/methyl groups at specific ring positions, can shift the inhibitory profile among these targets.

**Figure 8:**
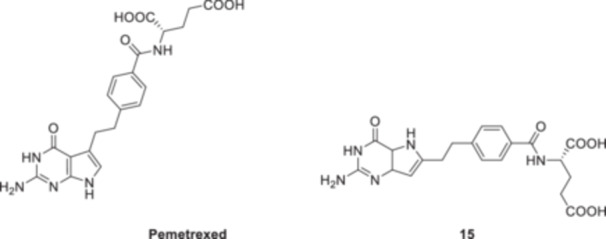
Pemetrexed and structure of **15**, a potent TS and DHFR inhibitor.

In 2017, *Tian* et al. designed, synthesized, and biologically tested novel 6‐substituted pyrrolo[3,2‐*d*]pyrimidines (Figure 8) as antifolate agents for cancer therapy [32]. These compounds are structurally similar to the thymidylate synthase (TS) inhibitor pemetrexed [33]. The compounds were tested on HL60 (human leukemia), A549 (human lung adenocarcinoma epithelial cells), H1299 (human non‐small cell lung carcinoma), HeLa, HCT116 (male human colon carcinoma), and HT29 (female human colon adenocarcinoma) cancer cell lines. Most of these compounds were active at micromolar concentrations. Compound **15**, the best among these compounds, showed a modest percentage of inhibition (67% at 100 µM) of DHFR. This may suggest that DHFR inhibition could not be its primary mechanism of action. Other experiments confirmed that adding extra thymidine could partially counteract the effects of **15**, reinforcing the idea that the compound mainly targeted TS. Notably, pemetrexed is known to act primarily on TS rather than DHFR, and thus, the focus was placed on its interaction with TS. Modeling and enzymatic assays confirmed that **15**, bearing a benzoyl‐glutamate tail, interacts with TS similarly to pemetrexed by forming critical hydrogen bonds with Asp218, Ala312, and Asn112. Unlike pemetrexed, the side chain of this compound is located at position 6 instead of position 5 of the pyrrolopyrimidine, in which the NH of the pyrrole is “relocated” to position 5 instead of 7. In DHFR, its binding closely resembled that of methotrexate (MTX, Figure 10) but with fewer hydrogen bonds. Specifically, the reported docked pose shows that the pyrrolo[3,2‐*d*]pyrimidine scaffold of **15** forms only two hydrogen bonds with Glu30, through the N3 and 4‐oxo groups, highlighting their essential role. In contrast, three hydrogen bonds were observed for MTX. Furthermore, the benzoylglutamate tail of **15** forms four hydrogen bonds with Gln35, Arg70 and Asn64, which are the same as those observed for MTX.

These results suggest that **15** may be a DHFR inhibitor, but is likely less potent than MTX which may explain its moderate, but still significant, activity. Compound **15** showed GI_50_ values of 0.7, 1.7, and 8.9 µM in A549, H1299, and HL60 cell lines, respectively. Cell cycle distribution analyses revealed that compound **15** increased the accumulation of cells in G2/M‐phase. In particular, while **15** did not induce apoptosis, it inhibited the formation of A549 cell colonies. This suggests that tumor cell death may depend on the sustained suppression of clonogenicity and proliferation by the compound, highlighting promising multi‐target antifolate activity [32].

Another investigation dates back to 2008, when *Gangjee* et al. discovered the antifolate compound **16** and various analogs as potential dual inhibitors of TS and DHFR, with a particular focus on cancer and parasitic infections [34]. In addition to the inhibition of human TS and DHFR, the authors were also interested in the selective inhibition of TS and/or DHFR of pathogens that cause opportunistic infections in HIV/AIDS patients, such as *Toxoplasma gondii*. Exploring substitutions at the N5 position of compound **16** allowed better hydrophobic interactions within human TS and DHFR enzyme pockets, thus improving potency.

Specifically, hydrophobic interactions were enhanced through the 6‐methyl group (molecular modeling indicates that this substituent makes crucial hydrophobic contacts with Trp109 in human thymidylate synthase), and the switch from the pyrrolo[2,3‐*d*]pyrimidine system (as in pemetrexed) to the pyrrolo[3,2‐*d*] one. The replacement of N7 with a carbon atom (C7) establishes a productive hydrophobic contact with Phe31 in human DHFR.

**Figure 9:**
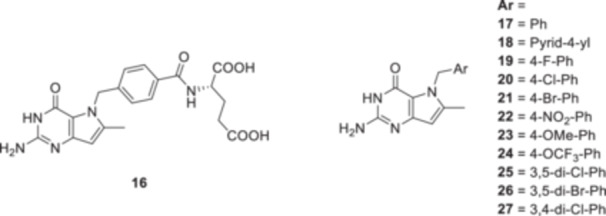
General structures of potential TS and DHFR dual inhibitors.

Compound **16** displayed potent dual inhibition of human TS (IC_50_ = 46 nM, approximately 206‐fold more potent than pemetrexed) [33] and DHFR (IC_50_ = 120 nM, about 55‐fold more potent than pemetrexed), largely attributed to favorable hydrophobic interactions. Extending these modifications to nonclassical analogs (compounds **17–27**), by adjusting the N5 substituents, it was possible to fine‐tune the dual inhibition of TS and DHFR. These compounds exhibited limited human TS inhibition, but four analogs (**20**, **21**, **22**, **24**) demonstrated strong inhibition of *Toxoplasma gondii* DHFR, with over 100‐fold selectivity compared to human DHFR (Figure 9) [23, 34].

**Figure 10:**
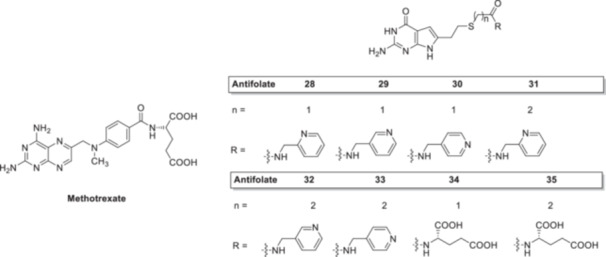
Methotrexate and antifolate compounds **28**–**35**.

In 2016, *Yi Liu* et al. reported a series of 6‐substituted pyrrolo[2,3‐*d*]pyrimidine compounds (Figure 10) structurally related to those reported by *Tian* et al., in which the benzamide moiety was replaced with a sulfur‐containing linker of variable length [35]. Side‐chain variations, including chain length and the position of the pyridine nitrogen, were found to influence antifolate potency and selectivity toward folate‐dependent enzymes. Another important structural difference is that these compounds belong to the class of pyrrolo[2,3‐*d*]pyrimidines like pemetrexed, while those reported 1 year later by *Tian* et al. and discussed above are all pyrrolo[3,2‐*d*]pyrimidines.

These derivatives showed micromolar to submicromolar antiproliferative activity against KB (human epithelial carcinoma), SW620 (colorectal cancer cells) and A549 cell lines. In particular, antifolate **35** (a 6‐substituted thioether) demonstrated notable potency (IC_50_ = 0.71 µM) in A549 cells, more effective than methotrexate (Figure 10) or pemetrexed (Figure 8) [36]. Studies on the molecular mechanism revealed that **35** was a dual inhibitor of TS and AICARFTase (5‐AminoImidazole‐4‐Carboxamide Ribonucleotide Formyl Transferase), while displaying reduced activity toward GARFTase (GlycinAmide Ribonucleotide Formyl Transferase). This suggests that the modification of the sulfur‐containing side chain is crucial for selectivity between GARFTase and AICARFTase. Supporting this hypothesis, mechanistic and modeling data indicated that the thioether functionality alters the electrostatic environment and binding geometry in the purine binding sites. Such selectivity is especially important because cancer cells exhibit an increased reliance on the *de novo* purine biosynthetic pathway and an impaired purine salvage pathway, whereas normal cells depend exclusively on the salvage pathway [37, 38]. Consequently, inhibitors targeting enzymes involved in de novo purine biosynthesis, such as GARFTase and AICARFTase, might selectively affect cancer cells while sparing normal cells. Furthermore, compound **35** was able to induce S‐phase accumulation and apoptosis in SW620 cells, leading to cell death. Altogether, these results highlighted the potential of compound **35** as a valuable starting point for developing more effective dual/multi‐target antitumor agents [35].

**Figure 11:**
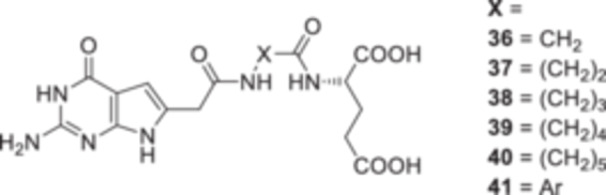
General structure of folate‐dependent enzymes' inhibitors **36–41**.

One year later, *Ruijuan Xing* et al. reported a series of 6‐substituted pyrrolo[2,3‐*d*]pyrimidines, with or without benzoyl chain, as potential antitumor agents (Figure 11) [39]. Notably, removing the phenyl ring, as in compounds **36–40**, the anticancer activity was preserved or boosted in comparison to classical benzoyl‐containing antifolates such as pemetrexed (Figure 8). These compounds showed strong antiproliferative activity against KB, SW620 and MCF‐7 cancer cells, in particular displaying nanomolar to subnanomolar potency towards KB cells. Compound **36** demonstrated an exceptional IC_50_ of 0.078 nM in KB cell culture, which is approximately 140‐ and 1000‐fold lower than methotrexate (Figure 10) and pemetrexed, respectively. This enhanced potency was attributed to its ability to retain robust hydrogen‐bonding motifs and adopt an extended conformation that places the glutamate tail in an optimal orientation. The benzoyl‐containing antifolate **41** (Figure 11) induced early apoptosis and cell‐cycle arrest in the S‐phase, leading to cell death. Both **36** and **41** effectively oriented the pyrrolo[2,3‐d]pyrimidine core within the TS, GARFTase, and AICARFTase, forming hydrogen bonds similar to those formed by pemetrexed. Furthermore, in‐depth analysis suggested that the replacement of the phenyl ring of **41** with a straight‐chain alkyl linker, as in derivatives **36–40** significantly increased antitumor activity. These results indicate that the aromatic group is not required for activity, and the optimal length of the alkyl amide linker between the pyrrolo[2,3‐*d*]pyrimidine core and the l‐glutamate is critical to achieve maximal inhibition (see Table 2) [39].

**Table 2:** SAR on Pyrrolo[2,3‐d]pyrimidine and pyrrolo[3,2‐d]pyrimidine‐based Compounds (1–60).

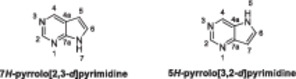				
Compound ID	Scaffold Modifications (SAR Summary)	Biological Target and Inhibitory Activity (IC_50_)		Computational Methods
**1f, 1n, 1l,1i,1c,1j**	**Sunitinib** core, **pyrrolo‐pyrimidine** scaffold; no C‐2/C‐5/C‐6 substitution; 4‐position long side‐chain modification. **1f** (4‐NMe_2_‐phenyl), **1n** (thiophene), **1l** (4‐CN‐phenyl): from the basic, H‐bonding small EDG to the compact heteroaromatic with low steric hindrance, and the EWG with similar sterics, all confer inhibitory activity against EGFR, HER2, VEGFR‐2, and CDK2. **1i** (3,4,5‐tri‐OMe‐phenyl): bulky EDG, high steric/electronic load → moderate HER2/VEGFR‐2 activity only. **1c** (4‐OH‐phenyl), **1j** (3‐OMe/4‐OH‐phenyl): the small polar **1c** yields only weak VEGFR‐2 activity, while the mixed donor/acceptor character of **1j** results in improved VEGFR‐2 activity. Compounds **1f** and **1n** showed excellent selectivity against the tested cancer cell lines (HepG2, HeLa, MDA‐MB‐231 and MCF‐7) and suppressed cell cycle progression of HepG2. Compound **1l** showed moderate selectivity and lower cellular activity.	**EGFR**: ▪ 0.15 µM for **1f** ▪ 0.03 µM for **1n** ▪ 0.07 µM for **1l** **HER2**: ▪ 0.04 µM for **1f** ▪ 0.03 µM for **1n** ▪ 0.04 µM for **1l** ▪ 0.41 µM for **1i**	**VEGFR‐2**: ▪ 0.03 µM for **1 f** ▪ 0.07 µM for **1n** ▪ 0.09 µM for **1 l** ▪ 0.19 µM for **1i** ▪ 0.30 µM for **1c** ▪ 0.05 µM for **1j** **CDK2**: ▪ 0.40 µM for **1 f** ▪ 0.13 µM for **1n** ▪ 0.43 µM. for **1 l**	Docking and in‐silico ADMET [19]
**2–7**	**Semaxanib** core; **pyrrolo‐pyrimidine** scaffold: no C‐5 substitution; C‐2 substitution with NH_2_; C‐4 substitution with 3‐bromoaniline; 6‐position single‐carbon side chain modifications. ▪ Highest inhibitory activity: small ortho‐substituted, hydrophobic phenyl groups (2‐methylphenyl, **2**) or 2,5‐dimethoxyphenyl (**3**). ▪ Reduced inhibitory activity: phenyl‐halogenation (2‐chlorophenyl, **5**); 2,4‐dichlorophenyl derivative (**4**) is nearly inactive on VEGFR‐2 but showed highest inhibitory activity on EGFR. ▪ Fused rings are tolerated (1‐naphthyl, **6**), but changing the connection point (2‐naphthyl, **7**) completely abolishes activity, highlighting a strict conformational constraint against VGRF‐2 but moderate activity against VEGFR‐1. It showed good activity against EGFR. Compound **2** and **4** showed cytotoxicity against A431 cells.	**VEGFR‐2**: ▪ 0.25 µM for **2** ▪ 0.62 µM for **3** ▪ 28.11 µM for **4** ▪ 5.58 µM for **5** ▪ 5.08 µM for **6** ▪ > 50 µM for **7**	**EGFR**: ▪ 0.23 µM for **4** ▪ 1.24 µM for **7** **VEGFR‐1**: ▪ 19.2 µM for **6** ▪ 15.2 µM for **7**	Molecular modeling [22]
**8**	**Semaxanib** core; **pyrrolo‐pyrimidine** scaffold: no C‐2/C‐5/C‐6 substitution; C‐4 substitution with 3‐bromoaniline.	**AURKA**: 2 µM **EGFR**: 3.8 nM		Docking [26]
UNC2025	**UNC1062** core; **pyrrolo‐pyrimidine** scaffold: no C‐4/C‐6 substitution; C‐2 substitution with butylamine; C‐5 substitution with benzyl‐4‐methyl piperazine; N‐7 substitution with cyclohexanol.	**MER**: 0.74 nM **FLT3**: 0.80 nM		/
**9–14**	**Pyrrolo‐pyrimidine** scaffold: no C‐2/C‐6 substitution; C‐4 substitution with NH_2_; C‐5 substitution with 4‐phenoxyphenyl; N‐7 4‐cyclohexyl modification (*trans L*‐Leu **9**, *trans* Gly **10**, *trans L*‐ser **11**, *trans L*‐Phe **12**, *trans D*‐Phe **13**, *cis D*‐tyr **14**). ▪ Best dual inhibitors: incorporation of aromatic amino acids (Phenylalanine **13**, Tyrosine **14**) significantly enhances potency, leading to the most effective dual inhibitors. The d‐Tyrosine *cis* isomer is identified as the most potent dual inhibitor, benefiting from an additional stabilizing OH hydrogen bond. ▪ Lower Activity Dual Inhibitors: Non‐aromatic α‐amino acids in *trans* configuration show high HCK inhibitory activity while *cis* configuration generally shows reduced HCK potency due to unfavorable binding to the Asp348 pocket. Simple acyclic amine derivatives lack the necessary polar interactions for HCK binding, resulting in a significant loss of dual inhibitory activity. Compounds **9** and **14** exhibited strong cytotoxicity against MV4‐11 cells.	**HCK**: ▪ 4.4 nM for **9** ▪ 3.7 nM for **10** ▪ 1.8 nM for **11** ▪ 7.5 nM for **12** ▪ 18 nM for **13** ▪ 13 nM for **14**	**FLT3‐ITD**: ▪ 14 nM for **9** ▪ 28 nM for **10** ▪ 18 nM for **11** ▪ 24 nM for **12** ▪ 2.6 nM for **13** ▪ 5 nM for **14**	Docking [30]
**15**	**Pemetrexed** core; **Pyrrolo‐pyrimidine** scaffold (*N*‐5): no C‐7 substitution, C‐2 substitution with NH_2_, C‐4 substitution with carbonyl group, C‐6 substitution with N‐Benzoyl‐l‐glutamic acid. GI_50_ values of 0.7, 1.7, and 8.9 µM in A549, H1299, and HL60 cell lines.	**DHFR**: inhibition of 67% at 100 µM. Other experiments confirmed that adding extra thymidine could partially counteract the effects of **15**, reinforcing the idea that the compound mainly targeted TS.		Docking [32]
**16** **17–27**	**Pemetrexed** core**; Pyrrolo‐pyrimidine** scaffold (*N*‐5): no C‐7 substitution, C‐2 substitution with NH_2_, C‐4 substitution with carbonyl group, C‐6 substitution with CH_3_, *N*‐5‐position long side‐chain modification. ▪ Best dual inhibitor: N‐5 substitution with N‐Benzoyl‐l‐glutamic acid (**16**). ▪ Lower Activity Inhibitors: compounds **17**‐**27** share the same pyrrolo‐pyrimidine scaffold scaffold but lack the l‐glutamic acid tail, feauturing various lipophilic aryl groups directly attached or linked to the 5‐position nitrogen. These nonclassical analogs are significantly less potent against human TS and generally less active against human DHFR (4‐Cl‐Ph **20**, 4‐Br‐Ph **21**, 4‐NO_2_‐Ph **22**, 4‐OCF_3_‐Ph **23**).	**DHFR**: ▪ 46 nM for **16** ▪ 0.35 µM for **20** ▪ 0.27 µM for **21** ▪ 0.33 µM for **22** ▪ 0.3 µM for **24** ▪ µM range others	**TS**: ▪ 120 nM for **16** ▪ µM range **17**‐**27**	Molecular modeling [34]
**28–35**	**Pemetrexed** core; **Pyrrolo‐pyrimidine** scaffold: no C‐5 substitution, C‐2 substitution with NH_2_, C‐4 substitution with carbonyl group, C‐6 substitution with sulfur linker of variable length. ▪ Best dual inhibitor: presence of the l‐glutamate moiety and the 2‐carbon linker in the side chain (**35**). ▪ Lower Activity Inhibitors: Replacing the glutamate tail with a lipophilic pyridine ring (**28**–**33**) generally leads to lower antiproliferative activity. ▪ Extending the alkyl linker from 1 carbon (n = 1, **28**‐**30**, **34**) to 2 carbons (n = 2, **31**‐**33**, **35**) generally improves potency.	Paper does not report direct IC_50_ values for isolated enzymes TS and AICARFTase.		Docking [35]
		SAR based on cellular antiproliferative IC_50_ data (A549 cells), which serves as the primary measure of biological potency: ▪ 28.4 µM for **28** ▪ 4.81 µM for **31** ▪ 2.87 µM for **32** ▪ 1.20 µM for **34** ▪ 0.71 µM for **35**		
**36–41**	**Pemetrexed** core; **Pyrrolo‐pyrimidine** scaffold: no C‐5 substitution, C‐2 substitution with NH_2_, C‐4 substitution with carbonyl group, C‐6 substitution with alkyl amide chain of variable length. ▪ Best dual inhibitor: linker length (n = 1) is favorable; shortest alkyl amide chain (Compound **36**) surprisingly yields the highest activity, suggesting minimal distance is optimal for positioning the *L*‐Glutamate moiety. Compound **36** is the single most potent agent, followed by **40**. When l‐Glutamate side chain is preserved in the classical orientation (linked *via* para‐aminobenzoic acid derivative **41**), it enables essential polyglutamylation and active uptake *via* the RFC. ▪ Lower Activity Inhibitors: Intermediate alkyl chain lengths [n = 2 (**37**), 3 (**38**), 4 (**38**)] result in suboptimal positioning of the glutamate tail within the enzyme active sites (TS, GARFTase, AICARFTase), confirming that a defined “optimal distance” of the linker is essential for dual‐target engagement. IC_50_ values provided are based on cellular antiproliferative assays (KB cell line) and not on direct assays of isolated enzymes (TS, GARFTase, AICARFTase). The multi‐target profile is confirmed qualitatively *via* mechanistic and computational studies.	**KB cell line:** ▪ 0.0034 µM for **36** ▪ 0.046 µM for **37** ▪ 0.057 µM for **38** ▪ 0.052 µM for **39** ▪ 0.0084 µM for **40** ▪ 0.015 µM for **41**		Docking [39]
**42–47**	**Pemetrexed** core; **Pyrrolo‐pyrimidine** scaffold: no C‐6 substitution, C‐2 substitution with NH_2_, C‐4 substitution with carbonyl group, C‐5 substitution with thiophene linker of variable length. ▪ Best dual inhibitor: linker lengths of four (**45**) or five (**46**) carbons provide the necessary flexibility and spatial reach for the l‐glutamate tail to simultaneously engage critical residues in the active sites of both targets. ▪ Lower Activity Inhibitors: shortest [n = 1, (**42**)] and longest [n = 6, (**47**)] linkers, and the intermediate lengths [n = 2, (**43**) 3 (**44**)], result in a suboptimal fit. IC_50_ values provided are based on cellular antiproliferative assays. The SAR is highly dependent on transporter uptake: KB cells primarily utilize RFC, while WfR cells express the α(FRα).	**KB**: ▪ > 100 µM for **42** ▪ 13.0 µM for **43** ▪ 2.3 µM for **44** ▪ 0.052 µM for **45** ▪ 0.075 µM for **46** ▪ 0.29 µM for **47**	**WfR**: ▪ 0.62 µM for **42** ▪ 0.22 µM for **43** ▪ 0.14 µM for **44** ▪ 0.076 µM for **45** ▪ 0.053 µM for **46** ▪ 0.18 µM for **47**	/
**52–53**	**Pemetrexed** core; **Pyrrolo‐pyrimidine** scaffold: no C‐5 substitution, C‐2 substitution with NH_2_, C‐4 substitution with carbonyl group, C‐6 substitution with thiophene linker of variable length. **52**: Thiophene‐3‐carboxylic acid derivative, carbonyl moiety attached to C‐3 of the thiophene ring. **53**: Thiophene‐2‐carboxylic acid derivative, carbonyl moiety attached to C‐2 of the thiophene ring. IC_50_ values provided are values reported are derived exclusively from cellular antiproliferative assays (e.g., IGROV1, SKOV3 cells) and not from direct inhibition assays against isolated enzymes (e.g., TS, AICARFTase). Therefore, the SAR is primarily driven by the efficiency of cellular uptake *via* the FRα and PCFT trasporters:	**IGROV1**: ▪ 0.0076 µM for **52** ▪ 0.017 µM for **53** **SKOV3**: ▪ 0.016 µM for **52** ▪ 0.021 µM for **53**		/
**54–55**	**Ruxolitinib** core; **Pyrrolo‐pyrimidine** scaffold: no C‐2/C‐5/C‐6 substitution, C‐4 substitution with pyrazoline‐linker of variable length. **54**: n = 6 linker **55**: n = 6; *N*‐methylated analog Compound **54** most active against the acute myeloid leukemia cell line HL‐60, the erythroleukemia cell line HEL92.1.7, and the acute T‐cell leukemia cell line Jurkat.	**HDAC1**: ▪ 7 nM for **54** ▪ 6 nM for **55** **HDAC6**: ▪ 1.4 µM for **54** ▪ 1 nM for **55**	**JAK2**: ▪ 75 nM for **54** ▪ 65 nM for **55**	Docking and Homology model [40]
**56**	**Ruxolitinib** core; **Pyrrolo‐pyrimidine** scaffold: no C‐5/C‐6 substitution, C‐2 substitution with homoallyl side chain, C‐4 substitution with pyrazoline‐linker.	**JAK2**: 41 pM **HDAC6**: 200 pM		Docking [41]
**57a–57b**	**Pyrrolo‐pyrimidine** scaffold: no C‐4/C‐5/C‐6 substitution, C‐2 substitution with pyrazoline‐ hydroxamate side‐chain, *N*‐7 substitution with 3‐fluorobenzyl moiety.	**JAK1:** ▪ 33 nM for **57a** ▪ 16 nM for **57b** **JAK2:** ▪ 33 nM for **57a** ▪ 20 nM for **57b** **JAK3:** ▪ 25 nM for **57a** ▪ 16 nM for **57b**	**HDAC1:** ▪ 34 nM for **57a** ▪ 92 nM for **57b** **HDAC6:** ▪ 8.4 nM for **57a** ▪ 14.7 nM for **57b**	Docking [42]
**58a–58b**	**Pyrrolo‐pyrimidine** scaffold: no C‐5/C‐6 substitution, C‐2 substitution with aniline side chain, C‐4 substitution with pyrazoline side‐chain.	**HDAC1:** ▪ 2.47 µM for **58a** ▪ > 100 µM for **58b** **HDAC6:** ▪ 0.008 µM for **58a** ▪ 6.30 µM for **58b**	**JAK2:** ▪ > 100 µM for **58a** ▪ 3.76 µM for **58b** **Hsp90α:** ▪ > 100 µM for **58a** ▪ 20.2 µM for **58b**	/
**59**	**Pyrrolo‐pyrimidine** scaffold: C‐2 substitution with amino group, C‐4 substitution with chlorine substituent, C‐5 substitution with benzyl‐hydroxamate for HDAC6 binding and N‐7 substitution with substituted pyridine for Hsp90 binding.	**HDAC6:**28 nM **Hsp90:** 96 nM		Docking [43]
**60 a–j**	**Pyrrolo‐pyrimidine** scaffold:	**ABCC1**	**ABCC1**	/
	**60a‐e**: no C‐2 substitution, C‐4 substitution with piperazine or phenyl side‐chain, C‐6 substitution with cyano moiety, pyrrolo ring fused to a six‐membered ring;	**calcein AM assay:** ▪ 0.37 µM for **60a** ▪ 0.67 µM for **60b** ▪ 2.56 µM for **60c** ▪ 5.06 µM for **60 d** ▪ 8.37 µM for **60e** ▪ 0.71 µM for **60 f** ▪ 1.06 µM for **60 g** ▪ 0.54 µM for **60 h** ▪ 0.24 µM for **60i** ▪ 0.75 µM for **60j**	**daunorubicin assay:** ▪ 0.20 µM for **60a** ▪ 0.64 µM for **60b** ▪ 2.90 µM for **60c** ▪ 2.87 µM for **60 d** ▪ 4.98 µM for **60e** ▪ 0.70 µM for **60 f** ▪ 0.79 µM for **60 g** ▪ 0.54 µM for **60 h** ▪ 0.25 µM for **60i** ▪ 0.34 µM for **60j** **ABCG2:** ▪ 8.19 µM for **60a** ▪ 8.24 µM for **60b** ▪ 5.91 µM for **60c** ▪ 4.25 µM for **60 d** ▪ n.t. for **60e–60j**	
	**60f‐j**: no C‐2 substitution, C‐4 substitution with piperazine linker, C‐6 substitution with cyano moiety, *N*‐7 substitution with side chain of different length. ▪ Best Balanced Dual Inhibitor **60a**: the phenethyl linker provides the necessary geometry and lipophilicity for high‐affinity ABCC1 binding, while the ABCG2 affinity is acceptable. ▪ Dual Hits with ABCG/ABCC1 Selectivity **60c** (phenil) & **60 d** (4‐methyl)): this suggests that ABCG2 requires a smaller or less rigid C‐4 side chain than ABCC1. ▪ Best ABCC1 inhibitor **60i**: optimal, flexible, and lipophilic group (cyclopropyl) at R_2_ position. Increasing aliphatic size at R_2_ did not correlate with increased activity.			

**Figure 12:**
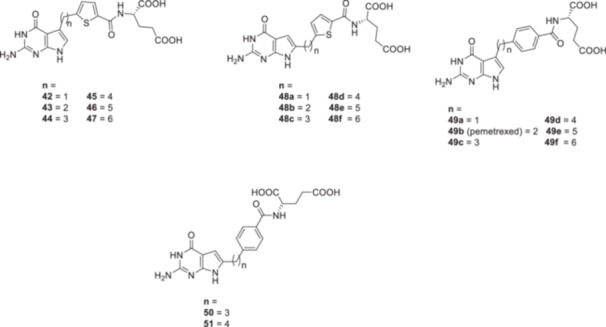
5‐ and 6‐substituted pyrrolo[2,3‐d]pyrimidines as potential antifolate agents.

In 2015, *Wang* et al. designed, synthesized and evaluated a novel series of 5‐alkyl‐thiophenyl substituted pyrrolo[2,3‐*d*]pyrimidines, with linker of different lengths (*n* = 1–6, compounds **42–47**, Figure 12). These compounds were assembled by combining the structure of pemetrexed (Figure 8) with that of previously reported 6‐thiophenyl substituted pyrrolo[2,3‐*d*]pyrimidines **48c** and **48d** [44, 45]. As already mentioned, the glutamate tail is crucial not only for enzyme binding but also for cellular uptake *via* folate transporters such as RFC, PCFT, and FRα. For instance, compounds **42–47** were specifically designed to target multiple transport pathways. They exhibited specific uptake *via* folate receptors (FRs) and proton‐coupled folate transporter (PCFT) over reduced folate carrier (RFC), with the best activity displayed by analogs containing 3‐ or 4‐carbon bridge. In contrast, the new 5‐substituted derivatives exhibited improved antiproliferative effects in RFC‐expressing cells, especially the analogs containing 2‐ and 4‐carbon bridge. However, they showed a pronounced loss of antiproliferative efficacy in FRα‐ and PCFT‐expressing cells for specific bridge lengths; such reduction was most severe for 4‐ and 5‐carbon analogs in FRα‐expressing cells, and for 2‐ and 5‐carbon analogs in PCFT‐expressing cells. Compounds **44**, **45**, and **46** inhibited purine nucleotide biosynthesis in the biochemical pathway involving GARFTase like the 6‐substituted analogs **48c, 48d** and the 6‐alkyl‐benzoyl substituted pyrrolopyrimidines **50** and **51**. Notably, derivatives **44** and **45** exhibited GARFTase and AICARFTase dual inhibition, evidenced by their impact on cell proliferation results. Similar results were obtained with **48c** and **48d**. In particular, compound **45** could access both RFC and FRα, in agreement with molecular docking predictions in which the glutamate tail and the thiophene ring adopt different orientations to accommodate optimally in each site. The 5‐alkyl‐benzoyl substituted pyrrolopyrimidines **49a–f** displayed a similar transporter specificity pattern compared with the 5‐alkyl‐thiophenyl derivatives, despite some differences in the impact of the bridge length. Moreover, **49c** and **49d** produced a significant depletion in ATP pools due to their combined inhibition of GARFTase and AICARFTase. The dual inhibition exerted by **44**, **45**, **48c** and **48d** may help maintaining antitumor efficacy in cases of resistance arising from mutations in either target. All these compounds hold great potential for further investigation. Variation from one to six carbons of the side chain underscored the importance of the chain's length and flexibility to gain a productive binding in FRα, PCFT, or RFC. Short linkers might restrict the molecule's conformational freedom, while excessively long ones can disrupt the optimal docking poses. The development of other analogs, aimed at optimizing cellular folate uptake through non‐RFC transport mechanisms, may improve tumor targeting and reduce toxicity [46].

**Figure 13:**
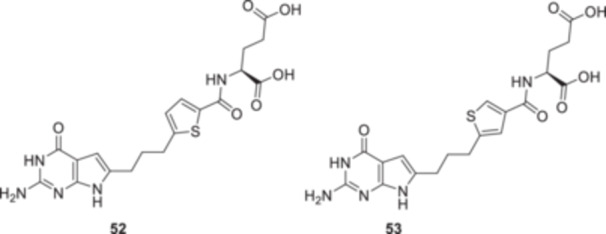
Structures of antifolates **52** and **53**.

In 2017, *Hou* et al. discovered a new generation of 6‐substituted pyrrolo[2,3‐*d*]pyrimidine‐thienoyl antifolates (**52** and **53**, Figure 13) endowed with antiproliferative activity and cytotoxicity against epithelial ovarian cancer (EOC) cells (IGROV1, SKOV3, A2780, and TOV112D). Specifically, compounds **52** and **53** displayed IC_50_ values ranging from 0.39 to 81 nM across these cell lines [47]. Their effectiveness is due to selective FRα and/or PCFT uptake, rather than that of RFC. It was observed that by treating IGROV1 (human ovarian cancer) cells with **52**, at slightly acidic pH (6.8), clonogenicity was significantly inhibited. In detail, researchers used FRα‐knocked down IGROV1 cells, and those with reduced FRα (KD‐4 and KD‐10 clones) showed less FRα‐dependent binding and uptake of [^3^H]folic acid and [^3^H]**53**, while high levels of PCFT‐dependent uptake of [^3^H]**53** were maintained compared with normal cells. In proliferation assays, **52** and **53** inhibited KD‐4 and KD‐10 cells in vitro. This result was attributed to the remaining FRα activity and significant uptake through PCFT. KD‐10 tumor xenografts derived from mice with compromised immune system were shown to be sensitive to **52**. All these findings highlight the therapeutic potential of these novel 6‐substituted pyrrolo[2,3‐*d*]pyrimidines as antifolate agents targeting both PCFT and FRα in EOC cells, including those resistant to cisplatin [47].

Here we have provided an overview on novel pyrrolopyrimidine‐based antifolates as potential antitumor agents. Many researchers have identified compounds that effectively inhibit the key folate enzyme/receptors such as TS, DHFR, GARFTase, AICARFTase, FR and PCFT. Examples include compounds **15**, **35**, **41**, **48**, and **49** illustrate how carefully designed structural changes such as core substitutions, modulation of side‐chain linker length, and different glutamate tails, are able to provide highly potent and selective antifolate agents with marked biological activity. Moreover, the ability of these compounds to induce apoptosis and cell‐cycle arrest along with their effectiveness against resistant tumor cell lines, underscores their potential for further preclinical studies.

For compounds **15–53**, visual SAR representations were not included because the folate‐related data are heterogeneous: some compounds are supported by target‐based data, whereas others are available only from cellular assays, which does not allow a consistent graphical representation. However, all compound data are provided in Table 2.

### 2.3 As JAK/HDAC Dual Inhibitors

**Figure 14:**
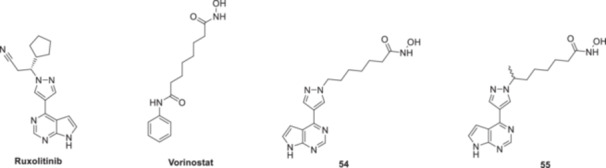
Ruxolitinib and vorinostat and their derivatives **54** and **55** as potent dual JAK/HDAC inhibitors.

Similar to their use in antifolate drug design, pyrrolo[2,3‐*d*]pyrimidines can be strategically functionalized to engage both Janus kinases (JAKs) and histone deacetylases (HDACs), two targets with very different binding sites. In JAKs, compounds typically form hydrogen bonds with key hinge residues (e.g., Glu930 and Leu932 in JAK2) and hydrophobic interactions with the glycine‐rich loop, allowing binding across JAK1/2 and, in some cases, JAK3 and TYK2. In HDACs, these inhibitors coordinate the catalytic zinc ion *via* a hydroxamic acid group, which could be reinforced by crucial hydrogen bonds with Tyr782 and His611 in HDAC6. The pyrrolo[2,3‐*d*]pyrimidine scaffold or appended substituents form complementary hydrophobic interactions near the entrance of the catalytic tunnel (the cap region). The dual inhibition of JAK and HDACs is particularly advantageous as it allows for the simultaneous modulation of inflammatory signaling pathways (*via* JAK inhibition) and epigenetic regulation (*via* HDAC inhibition), which may enhance therapeutic efficacy in diseases such as cancer and autoimmune disorders, where both processes play a crucial role. Indeed, in 2017, *Yao* et al. designed, synthesized and evaluated a new series of chimeric pyrrolopyrimidine ligands as dual JAK/HDAC inhibitors (Figure 14) [40]. Here, the core features of ruxolitinib [48], a marketed JAK1/2 inhibitor, have been merged with those of the HDAC inhibitor vorinostat [49], leading to new bifunctional targeted JAK/HDAC inhibitors. By optimization of the linker connecting the pyrazole and the hydroxamate group, compound **54,** with its pyrazolyl‐hydroxyheptanamide substituent, was identified as a potent derivative able to inhibit JAK1 and HDAC1, HDAC2, HDAC3 (Class I), HDAC6 and HDAC10 (Class IIb) with IC_50_ values of less than 20 nM. The hydroxamic acid coordinates the catalytic zinc in HDACs, while the pyrrolo[2,3‐d]pyrimidine ring forms hydrogen bonds with hinge residues in JAK1/2. In cellular assays, compound **54** simultaneously blocked JAK‐STAT signaling and induced hyperacetylation of histones and tubulin, thus reducing proliferation of multiple myeloma and AML cells. It showed IC_50_ values of < 100 nM against JAK2 and HDAC11 (Class IV) and, in general, it was demonstrated to be selective for the JAK family in a panel test with 97 kinases. However, the compound did not display selectivity among the different HDACs. Compound **54** was the most active against the AML cell line HL‐60 (7.36 µM), the erythroleukemia cell line HEL92.1.7 (1.33 µM), and the acute T‐cell leukemia cell line Jurkat (0.47 µM). Moreover, compound **54** was able to overcome tumor microenvironment–mediated resistance, enhancing tumor cell death, particularly in triple‐negative breast cancer models. It showed the highest potency against breast cancer cell lines MDA‐MB231 and MCF7, being the most sensitive with IC_50_ of 790 and 840 nM, respectively. Interestingly, low‐dose pharmacokinetic studies in rats indicated an oral exposure detectable for several hours after administration. Hence, given its low molecular weight, the high ligand efficiency and the good lipophilicity, **54** could be considered a promising lead compound to be further optimized. The methyl‐substituted analog **55** is more selective than **54**, with IC_50_ below 10 nM for HDAC1 and HDAC6 (6 nM and 1 nM, respectively). The IC_50_ values for HDAC 2, 3, and 10 were lower than 50 nM with much weaker inhibitory activity against other HDACs. Furthermore, **55** displayed increased cLogP and bioavailability. It is important to highlight that the goal of this work was to increase selectivity toward JAK1 and JAK2 while inhibiting class I and IIb HDACs, in order to maximize the anti‐proliferative effect while avoiding the inhibition of JAK3, which is involved in immune suppression. Importantly, both **54** and **55** exhibited low activity against JAK3, showing an IC_50_ value of 0.57 and 0.44 µM respectively [8, 40].

**Figure 15:**
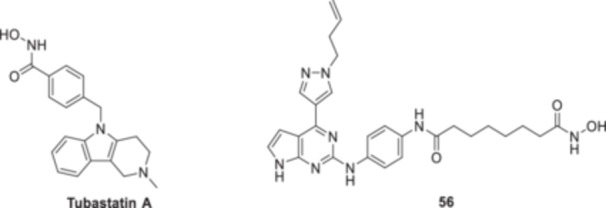
Tubastatin A and structure of **56** as JAK2 and HDAC6 dual inhibitor.

One year later, *Yao* et al. synthesized a new series of compounds that exhibited potent inhibition of JAK2 and HDAC6, with selectivity against HDAC1 [41]. One particularly promising compound, **56** (Figure 15), demonstrated unprecedented sub‐nanomolar potency against JAK2 (IC_50_ = 41 pM) and HDAC6 (IC_50_ = 200 pM).

**Figure 16:**
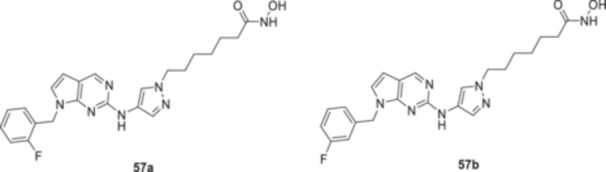
Structure of JAK2 and HDAC6 dual inhibitors **57a** and **57b**.

Moreover, **56** displayed antiproliferative activity in MCF‐7, MDA‐MB‐231 (human breast adenocarcinoma), HCT‐116 (human colon carcinoma), and PC‐3 (human prostate cancer) cell lines (with IC_50_ values ranging from 4.1 to 7.7 µM) compared with the selective HDAC6 inhibitor, tubastatin A (Figure 15) [50]. Computational binding models suggested favorable interactions with both JAK2 and HDAC6 active sites. Compound **56** bears a homoallyl side chain and a pyrazole substituent that fosters strong HDAC6 inhibition (hydroxamate head chelates the catalytic zinc, while the homoallyl‐pyrazole moiety engages in a shallow hydrophobic pocket around Thr678 and Met682) while retaining potent JAK2 binding through triple hydrogen bonds (e.g., Glu930, Leu932) [8, 41]. Another series of novel pyrrolo[2,3‐*d*]pyrimidine‐based derivatives, developed as potent dual inhibitors of JAK and HDAC, was discovered by *X. Liang* et al. in 2022 [42]. Notably, compounds **57a** and **57b** (Figure 16) possess a pyrrole ring and a hydroxamate side‐chain moiety, exhibiting strong inhibition of JAK1, JAK2, JAK3 and HDAC1, HDAC6 along with antiproliferative and proapoptotic effects in triple‐negative breast cancer cell lines. Molecular docking suggested that their effectiveness may arise from hydrogen bonds with Leu932 in JAK2 and robust zinc coordination in HDAC6. Modifications concerning the phenyl ring or side‐chain substitutions significantly influenced the potency and selectivity, offering clear structural points for optimization. These compounds reduced the activation of the LIFR‐JAK‐STAT (leukemia inhibitory factor receptor‐janus kinase‐signal transducer and activator of transcription) signaling pathway induced by tumor‐associated fibroblasts, indicating a potential efficacy in overcoming drug resistance driven by the tumor microenvironment. Compound **57a** effectively suppressed tumor growth in an MDA‐MB‐231 xenograft tumor model. This study highlighted novel antitumor mechanisms for addressing SAHA‐resistant triple‐negative breast cancers, offering valuable insights for further *in vitro* antiproliferative and in vivo preliminary antitumor activity assessments [8, 42].

**Figure 17:**
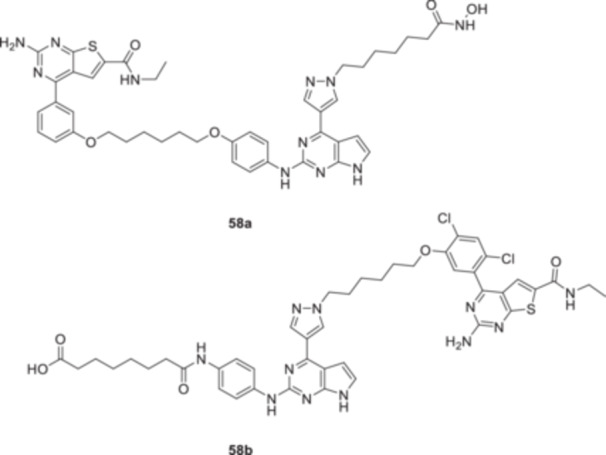
Compounds **58a** and **58b** as triple JAK2, HDAC6 and Hsp90 inhibitors.

In 2018, Yao et al. designed compounds aimed at inhibiting the three targets JAK2, HDAC and Hsp90, which are strongly implicated in oncogenesis [51]. Using the previously developed template for a JAK‐HDAC dual inhibitor [40], they synthesized a prototype of another dual inhibitor targeting both JAK and Hsp90. Initial testing revealed that some compounds exhibited low micromolar activity against JAK2, but moderate to no activity against Hsp90. Modifications, including the introduction of halogen substituents, improved Hsp90 inhibition in subsequent analogs. Compound **58a** (Figure 17) integrated an Hsp90 binding group with a JAK inhibitor scaffold while maintaining the HDAC hydroxamic acid, and demonstrated strong potency against HDAC6 but limited activity against the other targets. Compound **58b** (Figure 17), in which the HDAC‐binding moiety is linked to the JAK inhibitor's pyrimidine core and connected to the Hsp90 inhibitor's pyrazole ring, inhibited Hsp90, JAK2, and HDAC6 (IC_50_ = 20.2, 3.8, and 6.3 µM respectively). In particular, the Hsp90 inhibitory activity of compound **58b** is associated with the BEP800‐derived aryl substituent on the pyrimidine, while the pyrazole ring primarily contributes to HDAC inhibition. Although initial in vitro testing on compound **58b** revealed micromolar potency across all three targets, the partial inhibition of multiple disease pathways may offer preferable synergistic therapeutic effects with reduced toxicity compared with full inhibition of each target. All these findings clearly support the possibility to design small molecules capable of inhibiting multiple proteins critical for cancer progression, such as kinases, epigenetic targets, and chaperones [51].

Overall, these results highlighted the pyrrolo[2,3‐*d*]pyrimidine scaffold as a powerful tool for designing multifunctional inhibitors capable of simultaneously targeting JAKs and HDACs, with potential extension to triple‐target inhibition involving Hsp90. By strategically merging pharmacophores and carefully tuning substituents, these compounds reached sub‐nanomolar potency and good selectivity profiles, as well as promising antiproliferative effects against multiple cancer cell lines. Therefore, they represent a significant advancement in the discovery of anticancer drug candidates, particularly for hematologic malignancies and resistant solid tumors.

### 2.4 As Hsp90/HDAC6 Dual Inhibitors

**Figure 18:**
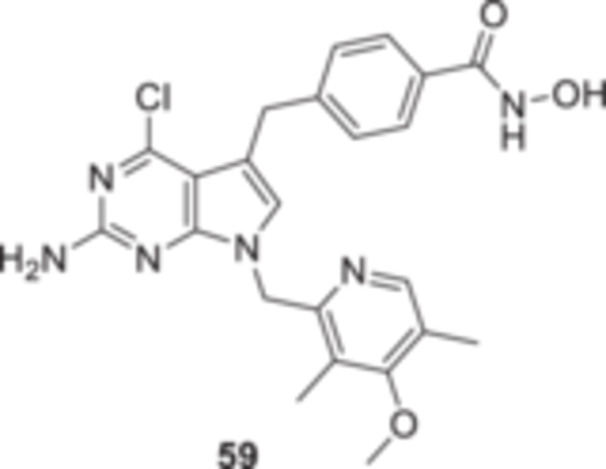
Compounds **59** as Hsp90/HDAC6 dual inhibitor.

In 2025, *Citarella* et al. reported the discovery of a potent dual inhibitor of Hsp90 and HDAC6 [43]. The compound was discovered through molecular modeling and structure‐based design into the binding pockets of the two enzymes. Despite the marked difference between the two binding sites, a potent and balanced nanomolar dual inhibitor was obtained (compound **59**, Figure 18) that showed potent antiproliferative activity in both 2D‐cultured and 3D tumor spheroid models of aggressive prostate cancer cell lines.

The compound showed favorable drug‐like properties and marked synergistic effects when tested in combination with doxorubicin. Therefore, this compound stands as a promising candidate for further preclinical evaluation against aggressive forms of prostate cancer.

**Figure 19:**
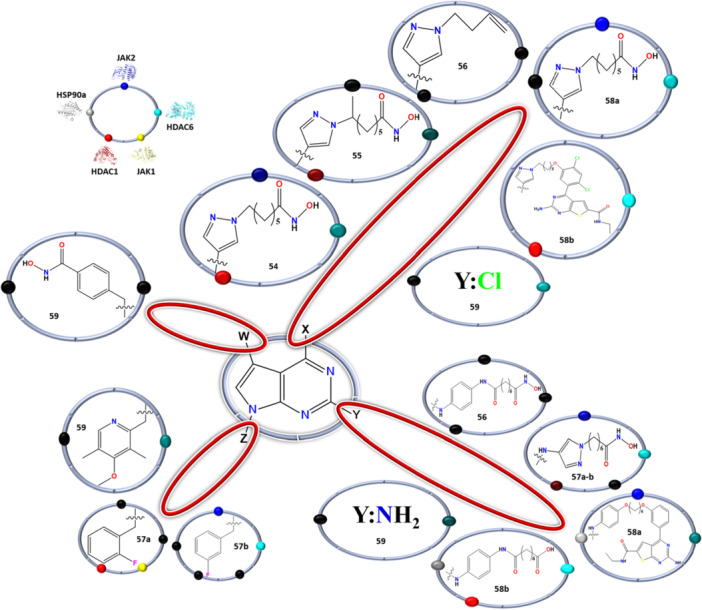
Third map of SAR for the pyrrolopyrimidine chemotype. The central panel shows the 2D core scaffold; red ovals denote structural determinants required for binding and inhibitory activity in the context of dual JAK/HDAC inhibition and Hsp90α targeting. Peripheral gray circles show the representative substituents explored with different substitution sites. For each substituent, colored dots indicate the specific target evaluated (HDAC1, red; HDAC6, cyan; Hsp90α, gray; JAK1, yellow; JAK2, blue) that has a fixed position on the gray circle. The compound's potency is encoded by darkening‐to‐black each colored dot (darker = higher potency), enabling rapid identification of multitarget profiles and relative strength against each target. Color assignment and position for each protein are depicted apart in the gray circle at the top left of the figure.

A visual overview of the SAR analysis for the investigated chemotypes **54**–**59** is provided in Figure 19.

### 2.5 As Inhibitors of Membrane Transport Proteins

**Figure 20:**
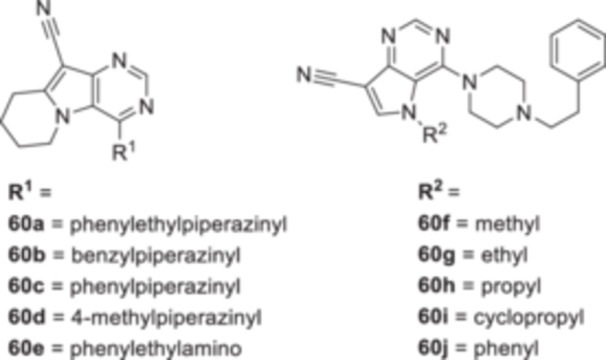
Structure of pyrrolopyrimidine derivatives as inhibitors of ABC transporters.

Membrane transport proteins, such as the ABC (ATP‐binding cassette) transporters, are crucial for the regulation of intracellular drug concentrations and drug efflux, making them important targets to overcome multidrug resistance. Due to their role in drug resistance, these proteins have attracted considerable interest in drug design. In some pyrrolo[2,3‐*d*]pyrimidine‐based derivatives, substitution at the 4‐position with a piperazine‐containing moiety, together with an additional lipophilic tail, plays a key role in modulating ABC transporter inhibition, especially toward ABCC1. In this context, *Wiese* et al. synthesized a series of pyrrolopyrimidine derivatives (Figure 20) and tested them against three ABC transport proteins (ABCB1, ABCC1, and ABCG2) [52, 53]. Most compounds exhibited stronger selectivity for ABCC1, the major target to overcome multidrug resistance in cancer patients. In particular, the authors investigated the effect of the aliphatic linker between the piperazine substructure at position 4 and the terminal phenyl ring, on ABCC1 inhibition and found that the replacement of phenethyl (**60a**) with a benzyl (**60b**) or phenyl (**60c**) reduced potency, suggesting that an ethyl group is preferable compared with methyl or no substitution. The removal of phenyl group (**60b**) led to compound **60d** with decreased activity and selectivity toward ABCC1, highlighting the importance of a lipophilic aromatic moiety for an effective transporter inhibition. Therefore, lipophilic groups, incorporating an aromatic ring moiety, were responsible for potent inhibition. The substitution of the piperazine (**60a**) with an amino group (**60e**) significantly reduced potency by over 22‐fold, underscoring the crucial role of the piperazine ring in achieving high‐affinity ABCC1 binding. Similarly, ring‐size variations such as from cyclohexyl to cycloheptyl negatively affected potency. Furthermore, substituting the cyclic system with aliphatic side chains at R_2_ afforded potent biological activity, with some compounds (**60a** *vs*. **60i**) having low micromolar IC_50_ values, indicating that flexible and lipophilic groups can maintain ABCC1 inhibition. However, increasing aliphatic size at R_2_ did not correlate with increased potency (**60f**, **60g)**. The replacement of the methyl (**60f**) with a phenyl group (**60j**) resulted in a comparable activity, suggesting acceptance of large aromatic residues at this position. In‐depth analysis highlighted that **60c** and **60d** displayed dual inhibition towards ABCC1 and ABCG2. The compounds were investigated in calcein AM (Calcein AcetoxyMethyl ester) and daunorubicin cell accumulation assays [54] using the doxorubicin‐selected and MRP1 overexpressing small cell lung cancer cell line H69 AR (antibiotic‐resistant). In these two biological assays, the reduction of ABCC1 (named also MRP1, multidrug resistance‐associated protein 1) overexpression and daunorubicin accumulation were respectively measured. The majority of the pyrrolopyrimidine derivatives proved to be non‐toxic, indicating that the reduction of daunorubicin resistance in H69 AR cells correlates with their ability to inhibit ABCC1 [53, 55].

Overall, these SAR studies emphasize the critical role of linker length, aromaticity, and lipophilicity of the substituent at position 4 on the pyrrolo[2,3‐*d*]pyrimidine scaffold, useful to achieve a potent ABCC1 inhibition. The versatility of these derivatives not only allows targeted inhibition of individual transporters but also provides opportunities for dual inhibition, with a consequent impact in overcoming drug resistance. Notably, these findings improved our understanding of the structural requirements for inhibiting ABC transporters in multi‐target contexts.

**Figure 21:**
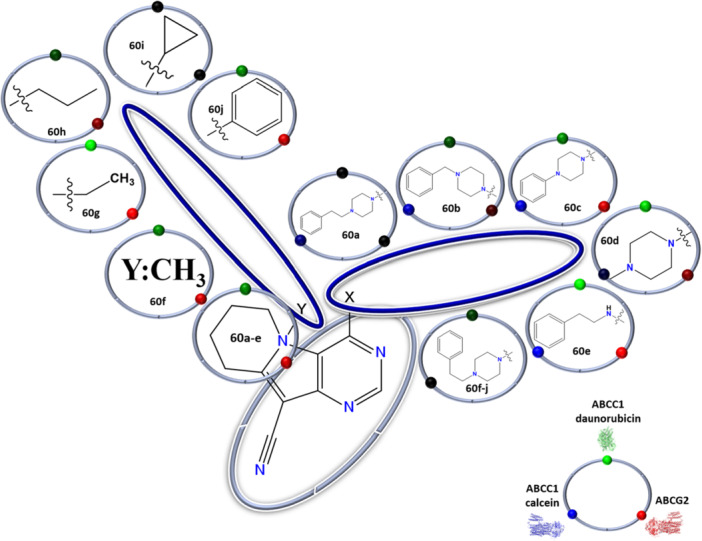
Fourth map of SAR for the pyrrolopyrimidine chemotype. The central panel shows the 2D core scaffold; blue ovals denote structural determinants required for binding and inhibitory activity of membrane transport proteins. Peripheral gray circles show the representative substituents explored with different substitution sites. For each substituent, colored dots indicate the transporter evaluated (ABCC1 (calcein‐AM) in blue; ABCC1 (daunorubicin) in green; ABCG2 in red) that has a fixed position on the gray circle. The compound's potency is encoded by darkening‐to‐black each colored dot (darker = higher potency), enabling rapid identification of multitarget profiles and relative strength against each transporter. Color assignment and position for each protein are depicted apart in the gray circle at the bottom right of the figure.

A visual overview of the SAR analysis for the investigated chemotypes **60** is provided in Figure 21.

### 2.6 SAR—Pyrrolo[2,3‐*d*]Pyrimidine Derivatives (1‐60)

Table 2 summarizes the SAR on the pyrrolo[2,3‐d]pyrimidine and pyrrolo[3,2‐*d*]pyrimidine derivatives (Compounds **1**–**60**) discussed above, condensing the activity results towards multiple targets, including protein kinases, folate enzymes, and ABC transporters.

## 3 Purines

### 3.1 As Dual Kinase Inhibitors

**Figure 22:**
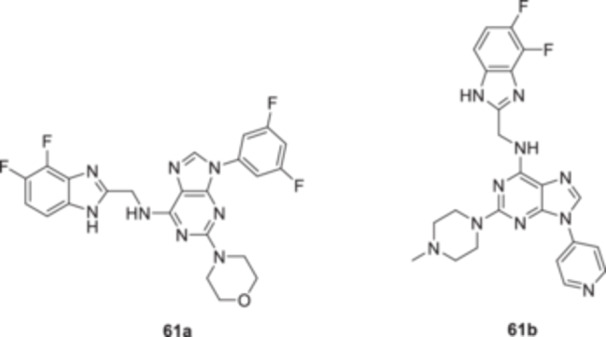
Structure of CK1 δ/ε dual inhibitors **61a** and **61b**.

The purine scaffold has proven versatile in targeting multiple kinases, ranging from casein kinase 1 isoforms (CK1δ/ε), Bcr‐Abl and Btk tyrosine kinases, and tyrosine kinase receptors (e.g., EGFR, HER2). By modifying substituents at the purine N9, C2, or C6 (sometimes combining ring‐fused moieties or additional aryl/heteroaryl groups), the obtained ligands exploit hydrogen‐bond interactions with the hinge region and hydrophobic interactions in additional subpockets. This approach has yielded potent and pharmacologically relevant dual inhibitors, some of which have antiproliferative activity against difficult‐to‐treat solid tumors and leukemias. SAR studies provided insights into the substituent patterns influencing multi‐target profiles. Building on these insights, *Monastyrskyi* et al. focused on developing dual inhibitors targeting casein kinase 1 (CK1) δ/ε to address cell metabolism in breast cancer treatment [56]. Dual inhibition of casein kinase 1 (CK1) δ/ε offers significant advantages in the treatment of cancer, neurodegenerative disorders, and cardiovascular diseases. CK1 is involved in regulating cell cycle, signaling pathways, and microtubule stability. By targeting both CK1δ and CK1ε it is possible to disrupt multiple key mechanisms that drive disease progression. Compounds **61a** and **61b** (Figure 22) showed strong inhibitory activity (IC_50_ = 53 nM and 175 nM, respectively) and effectively targeted the human MDA MB‐231 triple negative breast cancer cell line with EC_50_ values of 68 nM and 28 nM, respectively. **61a** and **61b** were further evaluated using both in vitro and in vivo assays, demonstrating favorable ADME properties. Based on these promising results, the compounds underwent in vivo PK testing. Molecular modeling studies indicated that these compounds interact with CK1δ *via* hydrogen bonds with Leu85. Substitution at purine N9 position significantly impacts potency: while alkyl groups markedly reduce activity, aromatic substituents such as a difluoro‐phenyl group retain low‐nanomolar activity. Introducing heterocyclic substituents, such as the benzimidazole moiety, substantially enhances potency; however, such modifications demand careful optimization to maintain adequate cell permeability. At the purine C2 position a morpholine or piperazine substituent generally increases the CK1δ/ε inhibition in comparison with *N*‐methylpiperazine analogs. Further modifications, such as elongating the distance to the morpholine ring by two or three carbons, or substituting it with thiomorpholine, negatively affect cellular potency while maintaining on‐target inhibition. Compounds **61a** and **61b** were also investigated with cancer xenograft studies [1, 56].

**Figure 23:**
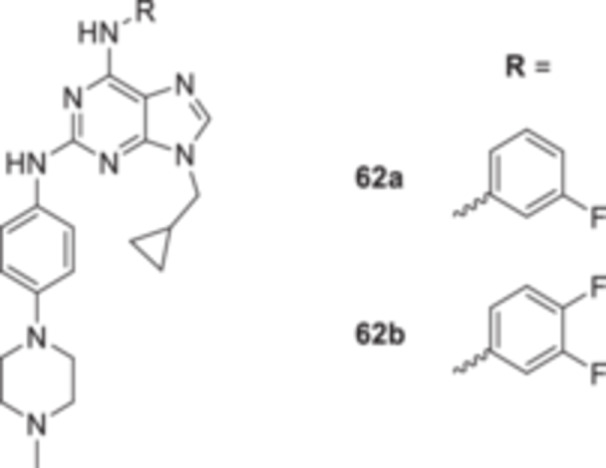
Structures of **62a** and **62b**, potent Bcr‐Abl and Btk kinase inhibitors.

Bcr‐Abl and Btk kinases are relevant pharmacological targets that play fundamental roles in the proliferation and survival of leukemia cells. *Bertrand* et al. developed tri‐substituted purine derivatives as Bcr‐Abl and Btk dual kinase inhibitors with the aim to reduce drug resistance to commonly used long‐term treatments. Among them, compounds **62a** and **62b** (Figure 23) showed similar inhibitory potency, with IC_50_ values of 40 nM for Bcr‐Abl and 0.58/0.66 µM for Btk, respectively.

SARs highlighted that fluorine substitution on the aniline ring, at meta‐ or meta‐ and para‐positions, increased Abl and Btk inhibitory activity, while a para‐fluorine substitution reduced potency. Moreover, fluorine substitution on the *N*‐methyl‐piperazinyl group at purine C2 decreased Bcr‐Abl and Btk inhibitory activity. Docking studies in Abl suggested π–π stacking interactions with aromatic residues such as Phe317 and Tyr320. Compound **62b** demonstrated robust inhibition of cells expressing the Bcr‐Abl protein. Compounds **62a** and **62b** suppressed cell proliferation of HL‐60, MV4‐11, CEM, K562, RAMOS and MCF‐7 cell lines. Compound **62a** showed GI_50_ values ranging from 0.77 to 5.77 μM, whereas compound **62b** exhibited GI_50_ values between 1.24 and 7.62 μM [1, 57].

**Figure 24:**
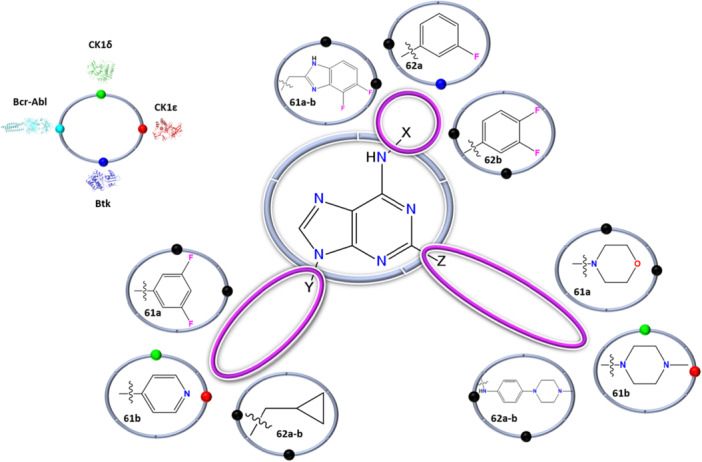
First map of SAR for the purine chemotype. The central panel shows the 2D core scaffold; purple ovals denote structural determinants required for binding and inhibitory activity of casein and tyrosine kinases. Peripheral gray circles show the representative substituents explored with different substitution sites. For each substituent, colored dots indicate the kinase evaluated (Bcr‐Abl, cyan; CK1δ, green; CK1ε, red; Btk, blue) that has a fixed position on the gray circle. The compound's potency is encoded by darkening‐to‐black each colored dot (darker = higher potency), enabling rapid identification of multitarget profiles and relative strength against each kinase. Color assignment and position for each kinase are depicted apart in the gray circle at the top left of the figure.

A visual overview of the SAR analysis for the investigated chemotypes **61**‐**62** is provided in Figure 24.

**Figure 25:**
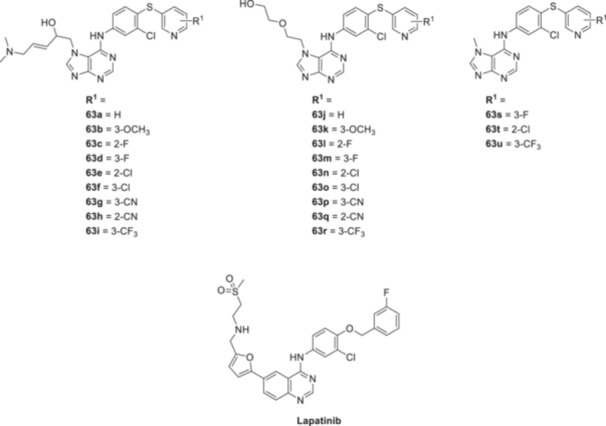
Lapatinib and structure of the kinase inhibitors.

In 2021, a new series of 6,7‐disubstituted‐7*H*‐purine derivatives was developed by *Chandraprakash Bayya* et al. aiming to arrest cancer growth through dual inhibition of EGFR and HER2 targets [58]. All compounds (Figure 25) underwent in vitro inhibition screening in a panel of seven kinases, including the four members of the EGFR family (EGFR, HER2, HER3, and HER4) as well as the tyrosine kinase receptors VEGFR, PDGFRα, and PDGFRβ (platelet‐derived growth factor receptor). Substituents at C6 and N7 significantly influenced potency. Introducing a 3‐chloro‐4‐(pyridin‐3‐yl)sulfanyl aniline at C6 position, combined with N7 substituents such as 5‐(dimethylamino)pent‐3‐en‐2‐ol or 2‐ethoxyethan‐1‐ol (compounds **63d**, **63e**, **63r**), markedly improved inhibitory activity. Five compounds (**63d**, **63e**, **63i**, **63m**, **63r**) were identified as the most potent EGFR inhibitors. In particular, the introduction of a trifluoromethyl‐pyridine substituent further improved efficacy, making compounds such as **63r** notably superior to lapatinib. Compound **63r** significantly inhibited EGFR and HER2 with IC_50_ of 0.017 and 0.014 µM respectively. Furthermore, in vitro cytotoxicity data highlighted the antiproliferative activity of **63r** in various breast cancer cell lines, as evidenced by its low GI_50_ (ranging from 1.54 to 3.20 µM, with the exception of MCF‐7), when compared with lapatinib (Figure 25) [59]. Moreover, **63i** and **63r** displayed promising IG_50_ values ranging from 1.68 to 2.24 µM against lapatinib‐resistant breast cancer cell lines BT‐474/L and SKBR‐3/L. In particular, treatment with **63r** resulted in selective downregulation of EGFR/HER2 dual kinase inhibition. Overall, these results suggest that modifying the purine core at positions 6 and 7 could lead to potential anti‐cancer agents particularly effective against lapatinib drug resistant forms [58, 60].

In light of these studies, the structural and pharmacological characteristics of various inhibitors have been investigated. In CK1δ/ε complexes, critical hydrogen bonds between the purine N1/N7 atoms and the hinge residue Leu85 were supported by additional stabilizing hydrophobic interactions involving benzimidazole or phenyl groups. In Abl/Btk complexes, key stabilizing contacts include hinge‐region hydrogen bonds (with M318 in Abl or M477 in Btk) and crucial salt‐bridge interactions mediated by positively charged piperazine groups. In EGFR/HER2 complexes, trifluoromethyl‐substituted derivatives typically engage in multiple hydrogen bonds with key hinge residues (Cys773 and Ala835 in EGFR; Ala751 and Asp808 in HER2) and π–π stacking interactions, consistent with their potent inhibitory profiles. From a pharmacokinetic perspective, several candidates demonstrated promising profiles, including moderate clearance (compounds **61a** and **61b**), suitable bioavailability, and tumor suppressive effects in xenograft models (compound **63r**). These results illustrate the versatility of purine scaffolds in designing potent inhibitors against multiple kinase targets.

**Figure 26:**
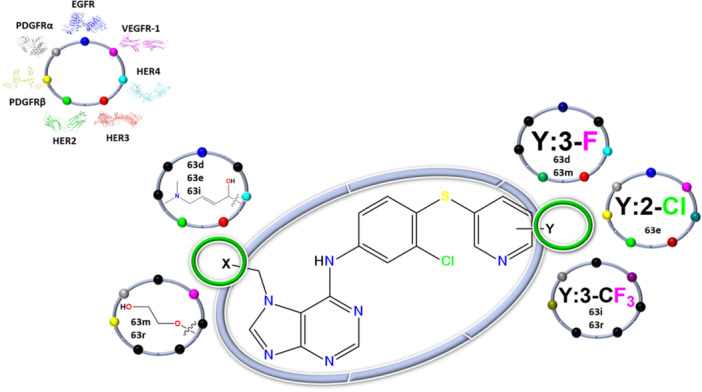
Second map of SAR for the purine chemotype. The central panel shows the 2D core scaffold; green ovals highlight substituents that are required for binding and inhibitory activity of tyrosine kinase receptors. Peripheral gray circles show the representative substituents explored with different substitution sites. For each substituent, colored dots indicate the receptor evaluated (EGFR, blue; HER2, green; VEGFR‐1, purple; HER3, red; HER4, cyan; PDGFRβ, yellow; PDGFRα, gray) that has a fixed position on the gray circle. The compound's potency is encoded by darkening‐to‐black each colored dot (darker = higher potency), enabling rapid identification of multitarget profiles and relative strength against each receptor. Color assignment and position for each receptor are depicted apart in the gray circle at the top left of the figure.

A visual overview of the SAR analysis for the investigated chemotypes **63** is provided in Figure 26.

### 3.2 As Inhibitors of HDACs and Kinases

Purine scaffolds functionalized with a zinc‐binding group (hydroxamate or benzamide) were engineered to inhibit both HDACs and kinases such as PI3K, mTOR, c‐Src, and CDK2. This dual approach may exploit synergistic mechanisms, for instance, by blocking HDAC‐regulated pathways that promote cancer cell survival, while also suppressing kinase‐driven proliferation signals. The resulting multitarget inhibition often produces synergistic antiproliferative effects against multiple cancer cell lines, including leukemias, lymphomas, and solid tumors (e.g., breast, liver, colon). HDAC inhibitors restore the expression of tumor‐suppressor genes, while PI3K inhibitors interfere with key signaling pathways that regulate cell survival, proliferation, and metabolism.

**Figure 27:**
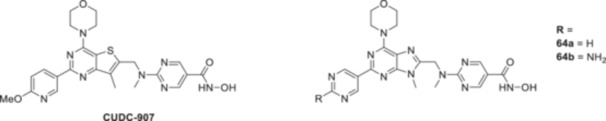
CUDC‐907 and structures of HDACs/PI3K dual inhibitors **64a** and **64b**.

In 2016, a new class of HDACs/PI3K inhibitors was developed by *Y. Chen* et al. by modifying the structure of CUDC‐907 (Figure 27). This molecule, discovered in 2013 [61, 62], is a potent inhibitor of HDAC1 (1.7 nM), HDAC2 (5.0 nM), HDAC3 (1.8 nM), HDAC6 (27.0 nM), HDAC10 (2.8 nM), and HDAC11 (5.4 nM) as well as PI3Kα (19 nM), PI3Kβ (54 nM), PI3Kδ (39 nM) and PI3Kγ (311 nM) [63]. The dual HDACs/PI3K inhibitors were designed and synthesized by merging the HDAC zinc binding group (i.e., hydroxamic acid) with the morpholino‐pyrimidine scaffold of CUDC‐907. This effort produced the purine analogs **64a** and **64b** (Figure 27). Replacing the phenyl moiety with an aminopyrimidine (e.g., compound **64b**) at position 2 of the morpholino‐purine core, the HDAC and PI3K inhibition were achieved with nanomolar potencies against PI3Kα and multiple HDAC isoforms. Compounds **64a** and **64b** showed potent HDAC1 inhibitory activity in comparison with SAHA and CUDC‐907, showing IC_50_ values of 1.14 nM and 1.05 nM, respectively. Compound **64b** also demonstrated an enhanced inhibition of PI3Kα, with an IC_50_ value of 1.33 nM, while removal of the amino group in **64a** resulted in lower activity (IC_50_ of 28 nM) [62, 63].

**Figure 28:**
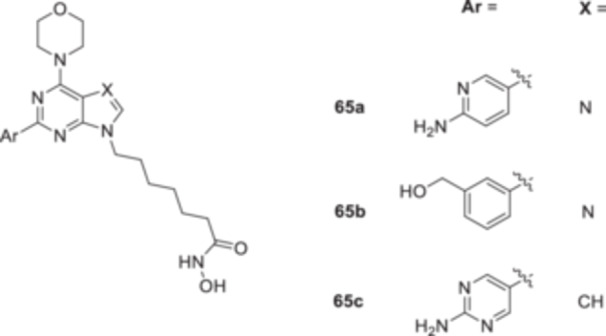
Structure of **65a–c**, potent HDACs and PI3K inhibitors.

In 2018, *D. Chen* et al. designed, synthesized, and biologically evaluated a new class of fused pyrimidine‐based hydroxamates to modulate both HDACs and the PI3K/Akt/mTOR (phosphoinositide 3‐kinase (PI3K)/protein kinase B (Akt)/mammalian target of rapamycin (mTOR)) pathway involved in the development of hepatocellular carcinoma (HCC) [64]. In particular, these compounds displayed dual HDAC1 and PI3K enzyme inhibition. Inhibiting HDAC1 can promote gene expression that regulates cell growth and survival, while targeting PI3K can disrupt signaling pathways involved in cancer progression. The introduction of a 2‐aminopyrimidine substituent at position 2 (compound **65b**) improved potency through key hydrogen bonding in the PI3Kα ATP pocket (residues Asp810, Tyr836, and Asp933), while the presence of a hydroxamic acid was required to achieve potent HDAC binding. Specifically, fused 2‐aminopyrimidine groups significantly enhanced the PI3Kα inhibitory activity. Compounds **65b** and **65c,** which contain a meta‐benzyl alcohol substituent, demonstrated dual or multi‐target inhibition by simultaneously inhibiting HDACs and PI3K/mTOR. Compounds **65a**, **65b**, and **65c** (Figure 28) were potent inhibitors of HDAC1, HDAC2, HDAC3, HDAC6, and HDAC10, but were less effective against classes IIa and IV HDACs. Compound **65b** and **65c** were effective against multiple kinases, with **65a**, **65b**, and **65c** also demonstrating selective inhibition of lipid kinases and promising activity in 39 cancer cell lines, as well as in MV4‐11 and HepG2 tumor models in mice. Specifically, **65b,** when administered orally, showed potent activity in mouse xenograft models of liver (HCC) and metastatic breast cancer (4T1). Further development of **65b** as a monotherapy or in combination with other drugs or in immunotherapy is warranted, particularly for leukemia (AML) and certain lymphomas such as diffuse large B‐cell lymphoma [8, 64].

**Figure 29:**
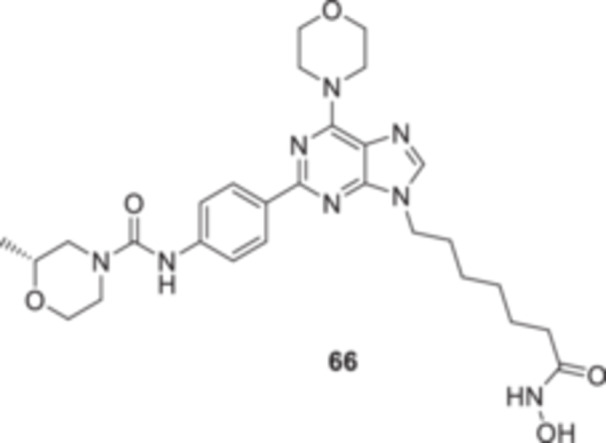
Structure of **66**, a HDAC1/mTOR dual inhibitor.

One year later, *Y. Chen* et al. synthesized dual inhibitors targeting mTOR and HDACs. The design approach exploited a pyrimidine‐pyrazolyl pharmacophore to incorporate the HDAC surface cap, and a hydroxamic acid as the zinc‐binding group. Among these compounds, **66** (Figure 29) presented a morpholine linked *via* a urea group to the purine scaffold and emerged as the most promising, exhibiting potent mTOR and HDAC1 inhibition with IC_50_ values of 1.2 nM and 0.19 nM, respectively. Moreover, **66** increased histone H3 and α‐tubulin acetylation and decreased mTOR mediators. In addition, **66** displayed strong activity against hematologic malignancy cells such as MV4‐11 and MM1S. Compound **66** induced cell‐cycle arrest in the G0/G1 phase and promoted tumor cell apoptosis. In a MM1S xenograft model, intravenous administration of **66** at a 20 mg/kg dose significantly inhibited tumor growth (tumor growth inhibition of 72.5%). Unfortunately, this compound had poor bioavailability. However, further compound optimization or a proper formulation could potentially overcome this limitation [8, 65].

**Figure 30:**
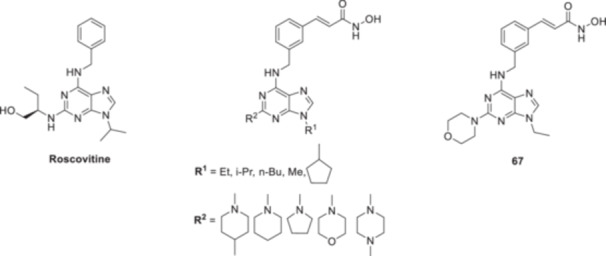
Structure of roscovitine and potent HDAC1/CDK2 dual inhibitor **67**.

In 2019, *Yu* et al. developed a series of purine derivatives as dual inhibitors of CDKs and HDACs by incorporating key pharmacophore elements taken from both classes. The best compound **67** integrated the zinc‐binding hydroxamic acid with a purine structure, resulting in IC_50_ values of 56 nM for CDK2 and 5.8 nM for HDAC1 (Figure 30).

**Figure 31:**
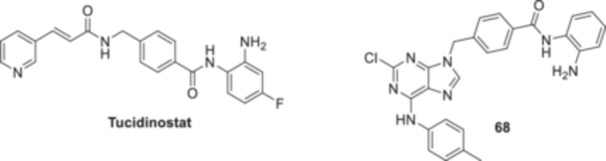
Tucidinostat and the HDACs/CDK2 inhibitor **68**.

This compound demonstrated apoptosis‐inducing effects against various cancer cell lines, particularly in HepG2 (IC_50_ = 0.77 µM). SAR analysis revealed that the morpholine group at R_2_ position contributed to increase CDK2 inhibition. On the contrary, other substitutions such as pyrrolidine, piperidine, 4‐methylpiperidine or *N*‐methyl piperazine reduced this activity. Moreover, large substituents at the R^1^ position were found to be detrimental, making the ethyl group the optimal one. Docking simulations suggested that the purine core of compound **67** accommodated in the ATP binding site of CDK2 similarly to roscovitine (Figure 30) [66]. Furthermore, like the isopropyl group in roscovitine, the ethyl group in R^1^ gave hydrophobic interactions with Ala144 and Phe80, and the hydroxamic acid made hydrogen bond interactions with Glu8 and Lys20, contributing to the higher potency of **67** compared with roscovitine [4, 67]. One year later, *Yun* et al. developed a series of novel inhibitors targeting CDK2 and HDACs enzymes. A purine‐based pharmacophore was integrated into the recognition cap group of tucidinostat, producing a library of compounds. The most potent of these, compound **68**, is shown in Figure 31. The compounds were evaluated for the antiproliferative activity toward five solid tumor cell lines, HCT116 (colon cancer), HepG2 (hepatocellular carcinoma), H460 (lung cancer), HeLa, A375 (malignant melanoma), in comparison with roscovitine and tucidinostat as reference compounds [68]. Compound **68** is a CDK2 (IC_50_ = 0.80 μM) and HDAC1 and HDAC2 (IC_50_ = 70.7 and 23.1 nM, respectively) inhibitor. The zinc‐binding group of this compound is represented by a benzamide moiety, as in tucidinostat, which is typical of selective class I HDAC inhibitors. Further pharmacological studies, including migration assays, indicated that **68** inhibited cancer cell migration, especially in H460 and A375 cell lines. **68** arrested cell‐cycle progression, particularly in the G2/M phase, and induced apoptosis in cancer cells. Intracellular ROS assay indicated that **68** strongly increased ROS levels in A375 cells, suggesting that this event might be responsible for cell death. Moreover, immunofluorescence assay demonstrated that compound **68** increased histone H3 acetylation, in agreement with the observed HDAC isoform selectivity profile. Importantly, the compound exhibited favorable pharmacokinetic data with an intraperitoneal bioavailability of 50.8% in mice and potent antitumor activity in a HCT116 xenograft model (TGI = 44%) [1, 69].

**Figure 32:**
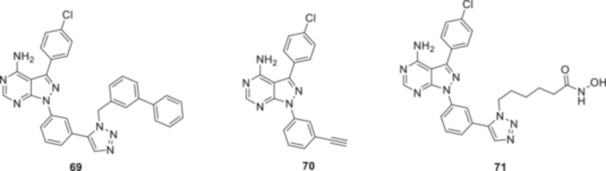
Structures of the c‐Src inhibitor **69**, precursor **70** and c‐Src/HDAC1 dual inhibitor **71**.

Another important target for the design of anticancer‐active kinase inhibitors is represented by Src (Sarcoma) tyrosine kinase. In 2013, *Kristin S*. et al. synthesized and evaluated new chimeric HDAC/Src inhibitors containing variable hydrophobic linkers. These compounds were further tested in combination with vorinostat, dasatinib, and the c‐Src (proto‐oncogene c‐Src) inhibitor **69** (Figure 32) to assess potential synergistic effects [70, 71]. Dasatinib is a dual Bcr‐Abl (breakpoint cluster region‐Abelson)/Src tyrosine kinase inhibitor, approved in 2006 for the treatment of chronic myeloid leukemia. To improve the Src‐inhibitory activity, the authors synthesized the alkyne precursor **70** that led to the triazole derivative **71** via a *click chemistry* synthetic protocol, combining structural elements of c‐Src inhibitors with a zinc‐binding hydroxamate group (Figure 32). Compound **71** demonstrated potent inhibition of both c‐Src (IC_50_ = 138 nM) and HDAC1 (IC_50_ = 0.26 nM) and exhibited strong activity against SK‐BR‐3 (IC_50_ = 0.2 µM) and MCF‐7 (IC_50_ = 0.35 µM) breast cancer cell lines [62, 72].

Docking studies of compound **71** confirmed key hydrogen‐bond interactions with PI3Kα residues (Val851, Asp810, Asp933, Tyr836, and Lys802 in the hinge region) and the characteristic HDAC zinc‐coordination with the hydroxamate. The PI3Kα is an intracellular kinase regulating cell growth, proliferation, and survival, often mutated in human cancers and is activated by receptor tyrosine kinases such as Src tyrosine kinase. Combining HDAC blockade with kinase inhibition induces robust apoptosis, S‐phase or G2/M cell‐cycle arrest. From an ADME perspective, many of these inhibitors showed rapid absorption but variable clearance rates. Nevertheless, compounds such as **65b** and **66** exhibited improved stability in human liver microsomes, positioning them as viable candidates for further development. In summary, these strategic structural modifications, particularly zinc‐binding groups combined with optimized kinase‐targeting substituents, yielded highly effective multitarget inhibitors (e.g., **65b**, **66**, **68**, **69**, **64b**). Optimizing the pharmacokinetics and solubility profiles will further enhance their clinical potential, offering valuable therapeutic options especially for aggressive and resistant types of cancer.

### 3.3 As JAK and BRD4 Dual Inhibitors

**Figure 33:**
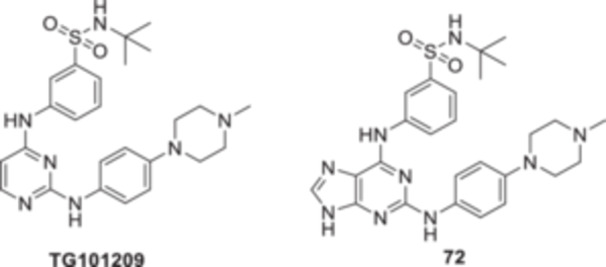
Structure of TG101209 and potent dual JAK2/BRD4 inhibitor **72**.

Two important targets for the treatment of myeloproliferative neoplasms (MPN) and inflammation are JAK2 and BRD4 (bromodomain‐containing protein 4). JAK2 drives cytokine signaling in hematological malignancies (e.g., JAK2 V617F mutation), while BRD4 regulates transcriptional activity through its bromodomains BD1 and BD2. Previous evidence suggested drug synergy [73] between JAK2 and BRD4 in the NF‐κB pathway (nuclear factor kappa‐light‐chain‐enhancer of activated B cells), indeed their combined effect enhances immune responses. In 2023 Yong Guo et al. developed a series of BRD4(BD2) and JAK2 dual inhibitors based on the structure of TG101209 (Figure 33) [74]. TG101209, bearing a tert‐butylsulfonamide‐aniline and a methyl‐substituted piperazine, is highly potent against JAK2 (IC_50_ = 6 nM) and inhibits BRD4 with an IC_50_ of 130 nM. They designed compounds with four different molecular scaffolds, and identified purine as the most effective one. Specifically, compound **72** (Figure 33) demonstrated moderate selectivity for BRD4(BD2) over BRD4(BD1) and emerged as an optimal candidate with acceptable selectivity toward the JAK kinase family. It possesses balanced activity against JAK2 and BRD4(BD2) with IC_50_ values of 13 and 22 nM, respectively.

**Figure 34:**
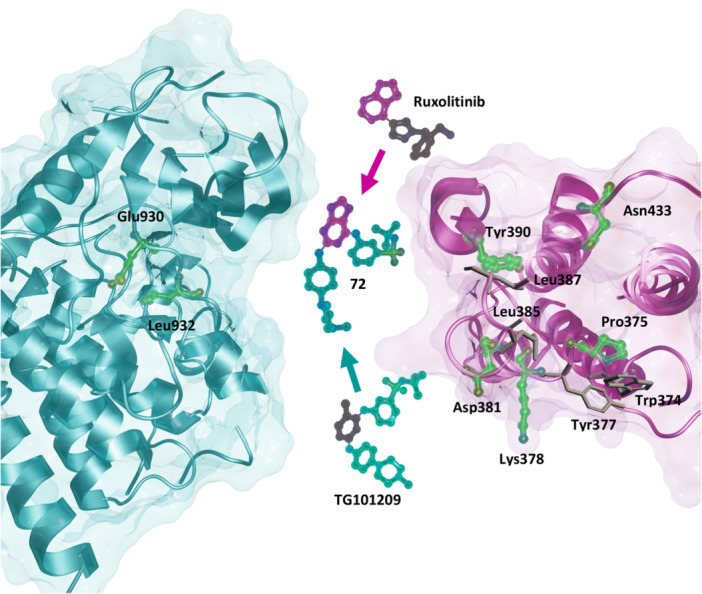
Structural representation of the dual‐target compound **72**, colored according to its structural hybridization from ruxolitinib (pink) and TG101209 (cyan), simultaneously bound to its protein targets JAK2 (cyan cartoon) and BRD4(BD2) (pink cartoon). Amino acids involved in hydrogen‐bond interactions are depicted in ball‐and‐stick style with carbon atoms colored in green, while residues mediating hydrophobic interactions are represented in licorice gray.

Extensive SAR analyses showed that moving from a pyrimidine to a purine scaffold markedly improved the activity toward BRD4(BD2) without compromising JAK2 potency. On the contrary, substitution of the pyrimidine with a pyrazolopyrimidine reduced activity toward both targets. Also, substituents on the aniline ring critically impacted dual inhibition. Alkoxy substituents in para‐position, especially methoxy or ethoxy groups, consistently improved dual JAK2 and BRD4 potency over electron‐withdrawing substituents (e.g., fluorine, trifluoromethyl). Additionally, para‐ substitution with polar aliphatic amines, such as morpholine or N‐methylpiperazine, dramatically increased the activity against BRD4 bromodomains and simultaneously enhanced the selectivity for JAK2 relative to other JAK family members (JAK1, JAK3, TYK2). Furthermore, the aniline's NH proved to be essential, as eliminating or methylating it significantly decreased dual activity. The imidazole NH was also essential for BRD4(BD2) engagement. Indeed, methylating or shifting this NH generally abolished or reduced dual activity. Based on these evidences, compound **72** emerged as particularly interesting since it combines the purine scaffold with optimized substituents such as the tert‐butylsulfonamide, the N‐methylpiperazine, and the para‐substituted aniline (Figure 34). Compound **72** exhibited strong cytotoxicity against hematologic malignancies, including Molm‐13 (human leukemia) and Ba/F3‐JAK2V617F (myeloproliferative neoplasms) cells. Western blot analysis confirmed that **72** effectively suppressed the NF‐κB signaling pathway and the phosphorylation of p65 and IκB‐α. Additionally, in in vivo ulcerative colitis model, the severity of the disease was alleviated by treatment with **72** (15, 30, and 60 mg/kg doses). Moreover, it exhibited high metabolic stability in human liver microsomes, making it suitable for further development. These results highlighted compound **72** as a promising lead compound targeting JAK2 and BRD4(BD2), which deserves further investigation for its potential therapeutic applications in treating myeloproliferative neoplasms and inflammatory diseases [73].

Detailed docking studies (Figure 34) provided mechanistic insights into the binding modes for both targets. In JAK2 (PDB: 4JI9), compound **72** adopts a binding orientation similar to TG101209, forming required hydrogen bonds with Leu932 and Glu930 in the hinge region. In BRD4(BD2), docking highlighted an intricate hydrogen‐bonding network with key residues such as Pro375, Lys378, Asp381, Tyr390, and Asn433.

Additionally, hydrophobic interactions with Trp374, Tyr377, Leu385, and Leu387 anchor the ligand within the bromodomain's acetyl‐lysine binding pocket, mimicking the typical interactions seen with BET (Bromodomain and Extra‐Terminal) inhibitors. This contributes to its strong affinity and selectivity toward BD2. Pharmacophore‐based models further improved understanding of the JAK and BRD dual inhibitory binding [75]. These pharmacophore models, combined with specific excluded volumes (spaces that the molecule cannot occupy because of steric hindrance) defining sterically restricted regions, serve as critical tools for ligand optimization and virtual screening, enabling exploration of chemical space beyond currently known scaffolds. Collectively, these findings underscore the therapeutic potential of purine‐based JAK2/BRD4 dual inhibitors, exemplified by compound **72**.

**Figure 35:**
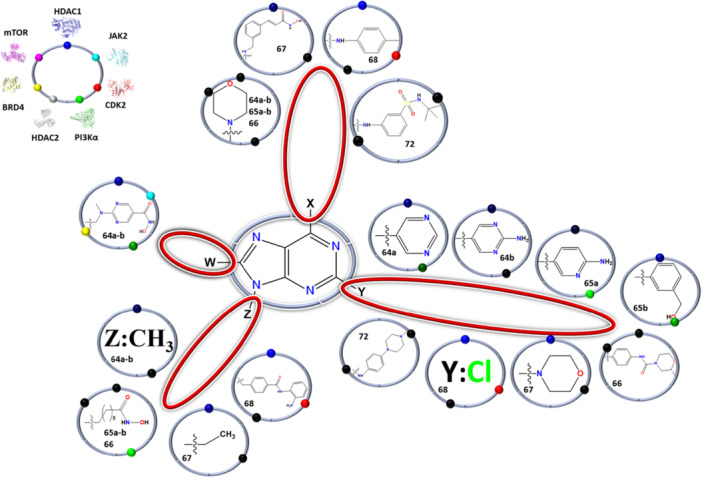
Third map of SAR for the purine chemotype. The central panel shows the 2D core scaffold; red ovals denote structural determinants required for binding and inhibitory activity in the context of HDAC and Kinase enzymes. Peripheral gray circles show the representative substituents explored with different substitution sites. For each substituent, colored dots indicate the enzyme evaluated (CDK2, red; JAK2, cyan; HDAC2, gray; BRD4, yellow; HDAC1, blue; mTOR, purple; PI3Kα, green) that has a fixed position on the gray circle. The compound's potency is encoded by darkening‐to‐black each colored dot (darker = higher potency), enabling rapid identification of multitarget profiles and relative strength against each enzyme. Color assignment and position for each enzyme are depicted apart in the gray circle at the top left of the figure.

A visual overview of the SAR analysis for the investigated chemotypes **64**‐**68** and **72** is provided in Figure 35.

### 3.4 As TRPA1 and PDE4B/7 A Inhibitors

**Figure 36:**
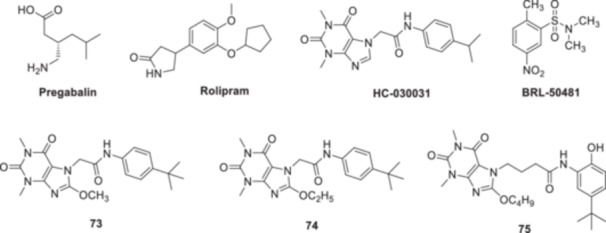
Structure of pregabalin, rolipram, HC‐030031, BRL‐50481 and **73**‐**75** as TRPA1 and PDE4B/7 A dual inhibitors.

In 2018, *Grażyna Chłoń‐Rzepa* et al. designed, synthesized and tested a new series of 1,3‐dimethyl‐2,6‐dioxopurin‐7‐ylalkylcarboxylic acid derivatives (Figure 36) [76].

By applying a molecular modeling‐assisted rational design, the authors designed multifunctional ligands acting as TRPA1 antagonists and PDE4B/7 A inhibitors for pain treatment [76]. TRPA1 is a calcium‐permeable ion channel involved in various pain and inflammatory processes, while PDE4B and PDE7A are cyclic nucleotide phosphodiesterases implicated in immune and inflammatory responses. In this work, phenylacetamide compounds **73** and **74** were found to be more effective against TRPA1 compared to HC‐030031 (Figure 36) [77]. Crucial substitutions on the phenyl ring significantly impact inhibitory activity: a 4‐*tert*‐butyl group enhances TRPA1 binding compared with substituents like 4‐isopropyl or 4‐chloro substituent. On the other hand, moving from the shorter acetamide linker to a longer butanamide, the TRPA1 potency generally decreases. However, incorporating lipophilic substituents (propoxy or butoxy) at position 8 partly compensates for this loss of potency, especially when such substitution is coupled with a 2‐hydroxy‐5‐*tert*‐butylphenyl group directly attached to the butanamide linker. Conversely, for PDE4B and PDE7A inhibition, the longer butanamide linker (compound **75**) (Figure 36) emerged as the most promising dual compound, acting both as a TRPA1 antagonist and a PDE4B/7A inhibitor. Within the butanamide series, the introduction of propoxy or butoxy substituents at position 8 of the purine core substantially improved the PDE inhibitory potency, achieving IC_50_ values ranging from 5 to 20 µM, comparable to known PDE4 or PDE7 reference inhibitors like rolipram and BRL‐50481 (Figure 36) [78, 79]. Again, the phenyl ring substitutions proved to be important, especially the 2‐hydroxy‐5‐*tert*‐butyl that markedly enhanced the PDE4B/7 A activity. Finally, **75** demonstrated potent anti‐inflammatory and pain‐relieving effects in animal models (such as the *formalin test* in mice and *carrageenan‐induced paw swelling* in rats). This compound also gave significant relief from allodynia in the early stages of chemotherapy‐induced nerve damage in mice. The effectiveness of compounds **73**, **74**, and **75** in alleviating allodynia was observed at doses comparable to those of the reference drug pregabalin (Figure 36) [80]. Additionally, **75** showed antiarthritic effects and a favorable pharmacokinetic profile in rats [81]. Docking studies provided mechanistic insights into ligand binding modes. In TRPA1, shorter‐linker compounds formed hydrogen bonds with Thr874 and π–π stacking with Phe877, in line with the binding mode of the reference antagonist HC‐030031. Butanamide derivatives, such as compound **75**, exhibited a distinct binding profile: the purine carbonyl at position 6 established key hydrogen bonds with Ser942 or Ser943, while the 2‐hydroxy substituent formed additional hydrogen bonds with Thr874. The presence of the bulky t‐butyl substituent increased hydrophobic interactions, further stabilizing ligand binding. As for PDE4B and PDE7A, docking simulations suggested that a four‐carbon butanamide linker is critical for the optimal positioning within the catalytic pocket of these enzymes. In PDE7A, compound **75** engaged in π–π stacking interactions with Phe416 and Phe384, while establishing hydrogen bonds with Gln413 and Glu282. Similarly, in PDE4B, **75** interacted through π–π stacking with Phe446 and Phe414, hydrogen bonds with Gln443 and, *via* its aryl substituent, His234/Asp392. These interactions may account for the PDE dual inhibition observed experimentally. Collectively, these studies illustrate the considerable potential of purine‐based scaffolds to simultaneously block TRPA1 channels and PDE enzymes. Specifically, compound **75** exemplifies how carefully optimized structural features, including linker length and purine and phenyl ring substitutions, can yield multifunctional agents with promising therapeutic efficacy to treat pain and inflammation.

### 3.5 As Toll‐Like Receptors (TLRs) Inhibitors

**Figure 37:**
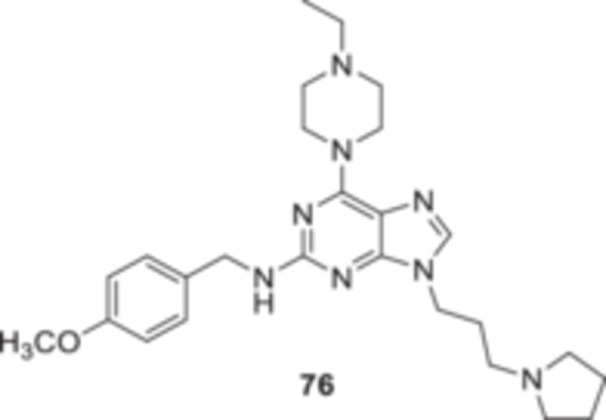
Structure of **76**, a potent TLR7 and TLR9 dual inhibitor.

TLRs are involved in immune and inflammatory responses and serve as crucial intracellular pattern‐recognition receptors (PRRs) that detect nucleic acids in endosomes, triggering interferon type I production and other proinflammatory pathways. In particular, dysregulated TLR7/9 signaling was implicated in autoimmunity (e.g., systemic lupus erythematosus, psoriasis). Purine scaffolds have been engineered to yield dual or selective TLR7/9 antagonists. These agents disrupt endosomal TLR activation, thereby dampening pathological immune responses. Potent dual TLR7/9 antagonists (Figure 37) were developed by *Biswajit Kundu* et al. in 2021 [82]. The SAR analyses elucidated critical structural modifications that influence TLR potency and selectivity. Substituents at the purine C2 position dramatically influence the behavior of compounds as TLR7/9 antagonists or agonists. Specifically, hydrophobic substituents such as benzylamines, particularly para‐substituted benzyl derivatives (4‐methoxybenzyl, 4‐fluorobenzyl, *N,N*‐diethylbenzyl), enhanced dual TLR7/9 antagonism (IC_50_ values often < 1 µM). In contrast, introducing smaller polar groups, such as amine or amide substituents, often shifted the selectivity toward TLR9 inhibition. Modifications at the C6 and at the N9 positions of the purine scaffold further improved the inhibitory profile. Considering the C6 position, piperazine substitution with aliphatic groups such as ethyl or cyclopentyl generally maintained or increased TLR7/9 antagonism. However, introducing electron‐withdrawing groups such as amides usually reduced or abolished TLR activity, suggesting the importance of balancing lipophilicity and basicity to gain an effective binding. At N9 position, flexible alkyl linkers (ethyl‐piperazine or propyl‐pyrrolidine) allowed better adaptability within the endosomal binding pockets. Bulky N9 substituents typically disrupt crucial binding interactions, whereas smaller moieties such as pyrrolidine favor dual‐target activity or improve TLR7 potency by maintaining optimal binding modes. Among these derivatives, compound **76** (Figure 37), incorporating a propyl‐pyrrolidine linker at N9 position and a 4‐methoxybenzylamine substituent at C2, showed a promising profile. This compound displayed potent dual inhibition of TLR9 (IC_50_ of 0.08 μM) and TLR7 (IC_50_ of 2.66 μM), sparing TLR8 up to a 30 μM concentration.

**Figure 38:**
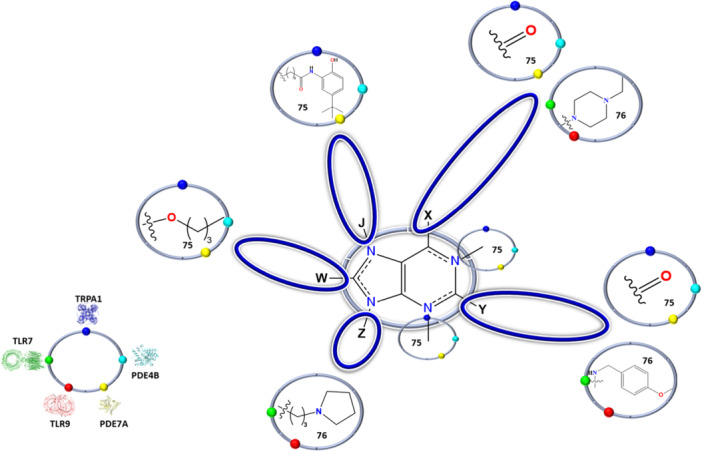
Fourth map of SAR for the purine chemotype. The central panel shows the 2D core scaffold; blue ovals denote structural determinants required for binding and inhibitory activity of TRPA1 ion channel, phosphodiesterases, and Toll‐like receptors. Peripheral gray circles show the representative substituents explored with different substitution sites. For each substituent, colored dots indicate the target evaluated (TRPA1 in blue; TLR7 in green; TLR9 in red; PDE7A in yellow; PDE4B in cyan) that has a fixed position on the gray circle. The compound's potency is encoded by darkening‐to‐black each colored dot (darker = higher potency), enabling rapid identification of multitarget profiles and relative strength against each target. Color assignment and position for each protein are depicted apart in the gray circle at the bottom left of the figure.

Docking studies in TLR7 homology models indicated that hydrophobic substituents bind in a hydrophobic pocket lined by residues Phe327 and Phe329, and critical hydrogen bond are formed with Ser452. Compounds with bulky substituents at C2 or N9 typically forced the purine scaffold into an unfavorable conformation (“flipped”), thus reducing critical hydrogen‐bonding and π–π interactions essential for TLR7 binding, favoring selective TLR9 antagonism. Because the antagonist‐binding region differs between TLR7 and TLR8, selectivity can be tuned: notably, Thr384 in TLR7 is replaced by the bulkier, nonpolar Ile403 in TLR8, while Ser452 (and Glu459) do not align with comparable residues in TLR8. Isothermal titration calorimetry (ITC) excluded direct interactions between these purine‐based antagonists and the TLR agonists CpGB and CL264, supporting the interpretation that their antagonistic activity is mediated by interaction with the corresponding TLR proteins. Compound **76** demonstrated potent selective TLR9 inhibition (IC_50_ 0.036 µM) along with moderate TLR7 antagonism. Its excellent in vitro pharmacokinetic properties included moderate‐to‐high solubility, robust stability in plasma and liver microsomes, high permeability (Caco‐2 cells), and favorable oral bioavailability (approximately 38% in mice). Importantly, in vivo pharmacokinetic studies highlighted that **76** has a suitable half‐life (3.4 h) and a good oral bioavailability, achieving systemic exposure above the TLR9 IC_50_, thereby supporting its therapeutic potential. Moreover, cytotoxicity assessments on various cell lines (PBMCs, HCT116, HepG2, H9c2) confirmed its favorable safety profiles. These findings support compound **76** as a promising candidate for future development into a clinically relevant molecule [82]. A visual overview of the SAR analysis for the investigated chemotypes **75–76** is provided in Figure 38.

### 3.6 SAR—Purine Derivatives (61–76)

Table 3 summarizes the SAR on the purine derivatives **61–76**.

**Table 3:** SAR on 9H‐purine‐based compounds **(61–76)**.

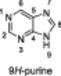				
Compound ID	Scaffold Modifications (SAR Summary)	Biological Target and inhibitory Activity (IC_50_)		Computational Methods
**61a–b**	**Purine** scaffold: no C‐8 substitution, C‐2 substitution with morpholine moiety (**61a**) or *N*‐methyl pyperazine (**61b**), C‐6 substitution with benzimidazole side chain, *N*‐9 substitution with 3,5‐difluorophenyle (**61a**) or pyridine (**61b**).	**CK1δ**: ▪ 53 nM for **61a** ▪ 145 µM for **61b**	**CK1ε**: ▪ 175 nM for **61a** ▪ 315 nM for **61b**	Docking [56]
**62a–b**	**Purine** scaffold: no C‐8 substitution, C‐2 substitution with aniline moiety, C‐6 substitution with 3‐fluoroaniline (**62a**) or 2,3‐difluoroaniline (**62b**); *N*‐9 substitution with cyclopropyl side chain.	**Bcr‐Abl**: ▪ 0.04 µM for **62a** ▪ 0.04 µM for **62b**	**Btk**: ▪ 0.66 µM for **62a** ▪ 0.58 µM for **62b**	Docking [57]
**63a–u**	**Lapatinib** core; **purine** scaffold: no C‐2/C‐8 substitution; C‐6 substitution with aniline side chain, *N*‐7 substitution with alkyl chain of different length. ▪ Best dual inhibitors: required trifluoromethyl group (**63i**) on the pyridine ring. N7 Tail: Large, polar/basic hydrophobic tail required. Compound **63r** (possesses a 2‐ethoxyethan‐1‐ol N7 tail) demonstrated the highest overall dual EGFR/HER2 potency. ▪ Lower Activity Dual Inhibitors: Substituted groups other than the electron‐withdrawing ‐CF_3_ (e.g., Cl or H on the pyridine ring) led to reduced potency compared to **63i** and **63r.** **N7 tail**: Substitution with a simple methyl group resulted in the complete loss of kinase inhibitory activity.	**EGFR**: ▪ 0.05 µM for **63 d** ▪ 0.09 µM for **63e** ▪ 0.03 µM for **63i** ▪ 0.07 µM for **63 m** ▪ 0.02 µM for **63r** **HER2**: ▪ 0.04 µM for **63 d** ▪ 0.07 µM for **63e** ▪ 0.02 µM for **63i** ▪ 0.05 µM for **63 m** ▪ 0.01 µM for **63r** **HER3**: ▪ 0.16 µM for **63 d** ▪ 0.09 µM for **63e** ▪ 0.06 µM for **63i** ▪ 0.09 µM for **63 m** ▪ 0.04 µM for **63r** **HER4**: ▪ 0.09 µM for **63d‐e** ▪ 0.08 µM for **63i**	**HER4**: ▪ 0.10 µM for **63 m** ▪ 0.06 µM for **63r** **VEGFR1**: ▪ 0.32 µM for **63 d** ▪ 0.78 µM for **63e** ▪ 0.57 µM for **63i** ▪ 0.52 µM for **63 m** ▪ 0.55 µM for **63r** **PDGFRα**: ▪ 0.21 µM for **63 d** ▪ 0.88 µM for **63e** ▪ 0.35 µM for **63i** ▪ 0.44 µM for **63 m** ▪ 0.64 µM for **63r** **PDGFRβ**: ▪ 0.27 µM for **63 d** ▪ 0.65 µM for **63e** ▪ 0.28 µM for **63i** ▪ 0.52 µM for **63 m** ▪ 0.66 µM for **63r**	/
**64a–b**	**CUDC‐907** core; **purine** scaffold: C‐2 substitution with pyrimidine (**64a**) or aminopyrimidine (**64b**) moieties, C‐6 substitution with morpholine group, C‐8 substitution with pyrimidine side chain, N‐9 methyl. ▪ Best dual inhibitor: **64b**; nanomolar potencies against multiple HDAC isoforms.	**HDAC1:** ▪ 1.14 nM for **64a** ▪ 1.04 nM for **64b**	**PI3Kα:** ▪ 28.1 nM for **64a** ▪ 1.33 nM for **64b**	Docking [63]
**65a–c**	**Purine** scaffold: no C‐8 substitution; C‐2 substitution with aminopyridine (**65a**), benzyl alcohol (**65b**), aminopyrimidine (**65c**, pirrolo‐pyrimidine scaffold), C‐6 substitution with morpholine, N‐9 substitution with alkyl chain of different length. They showed strong inhibition of HDAC 1, 2, 3, 6, and 10, but were less effective against classes IIa and IV.	**HDAC1:** ▪ 0.85 nM for **65a** ▪ 2.7 nM for **65b** ▪ 1.5 nM for **65c**	**PI3Kα:** ▪ 196 nM for **65a** ▪ 50 nM for **65b** ▪ 25 nM for **65c**	/
**66**	**Purine** scaffold: no C‐8 substitution; C‐2 substitution with phenyl methylmorpholine, C‐6 substitution with morpholine, N‐9 substitution with alkyl chain of different length.	**HDAC1**: 0.19 nM **mTOR**: 1.2 nM		Docking [65]
**67**	**Roscovitine** core**; Purine** scaffold: no C‐8 substitution, C‐2 substitution with morpholine, C‐6 substitution with side chain containing hydroxamic acid, N‐9 substitution with ethyl group.	**HDAC1**: 5.8 nM **CDK2**: 56 nM		Docking [67]
**68**	**Tucidinostat** core**; Purine** scaffold: no C‐8 substitution, C‐2 substitution with Cl, C‐6 substitution with aniline moiety, N‐9 substitution with side chain containing aniline.	**HDAC1**: 70.7 nM **HDAC2**: 23.1 nM **CDK2**: 0.80 µM		Docking [69]
**69–71**	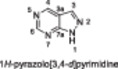	**HDAC1**: ▪ n.t. for **69** ▪ n.t. for **70** ▪ 0.26 nM for **71**	**Src**: ▪ 44 nM for **69** ▪ 1.45 nM for **70** ▪ 138 nM for **71**	/
	**Modified Purine** scaffold: no C‐6 substitution, C‐3 substitution with 4‐Cl benzene, C‐4 substitution with amine, N‐1 substitution with biphenil triazole (**69**), triazole containing an alkyl side chain (**71**) and benzyl (**70**) moieties. ▪ Best dual inhibitor: **71**.			
**72**	**TG101209** core**; Purine** scaffold: no C‐8 substitution, C‐2 substitution with piperazine moiety, C‐6 substitution with aniline moiety.	**JAK2**: 13 nM **BRD4(BD2)**: 22 nM		Docking [74]
**73–75**	**Purine** scaffold: N‐1/N‐3 methyl substitution, C‐2/C‐6 substitution with carbonyl group, C‐8 substitution with O‐substituted side chain, N‐7 substitution with different acetamide side chains. ▪ Best TRPA1 Antagonists: **73‐74.** Short (Acetamide). Phenyl Substituent: 4‐*t*‐Butyl (optimal), superior to 4‐isopropyl or 4‐chloro. ▪ Most Promising Dual Inhibitor: **75**. Long (Butanamide), critical for PDE activity. Purine Substituents: Lipophilic (OPr or OBu) at position 8, enhance PDE potency. Phenyl Substituent: 2‐OH‐5‐t‐Butyl, essential for boosting dual activity	**75:** **TRPA1**: 39 µM **PDE4B**: 5.43 µM **PDE7A**: 5.07 µM		Induced Fit and Docking [76]
**76**	**Purine** scaffold: no C‐6 substitution, C‐2 substitution with benzyl amine moiety, C‐8 substitution with ethyl piperazine, N‐9 substitution with pyrrolidine‐propyl side chain.	**TLR9**: 0.08 µM **TLR7**: 2.66 µM		Docking and Homology Model [82]

## 4 Discussion and Conclusions

The development of purine and pyrrolo[2,3‐*d*]pyrimidine derivatives as multi‐target therapeutic ligands represents a rational and increasingly attractive approach in medicinal chemistry, particularly for the treatment of complex diseases such as cancer. These heterocyclic scaffolds are widely recognized as privileged structures able to engage diverse biological targets, including protein kinases, folate enzymes, epigenetic regulators, membrane transporters, and immune‐related receptors, thus offering a versatile platform for the design of polypharmacological drugs. The multi‐target approach offers significant therapeutic advantages over single‐target strategies. By simultaneously modulating multiple biological targets, a single molecular entity may improve efficacy, mitigate drug resistance, and reduce the need for drug combination regimens that increase the risk of drug–drug interactions. Representative compounds discussed herein (e.g., **1n**, **3**, **8**, **35**, and **54)** exemplify this concept by showing dual inhibitory profiles, such as VEGFR‐2/CDK2 or JAK/HDAC isoforms, leading to potent antiproliferative activity across multiple tumor cell lines.

Clear SAR trends emerge across the reported series. Substitutions at the C‐2 position (e.g., amino or aryl groups) are critical for kinase engagement, forming key hydrogen bonds in the ATP‐binding hinge region. In pyrrolo[2,3‐*d*]pyrimidines, C‐4 aryl or heteroaryl groups (such as anilines or pyrazoles) enhance kinase selectivity and steric compatibility with allosteric pockets or gatekeeper residues, while C‐6 accommodates bulky or lipophilic moieties (e.g., benzyl or aralkyl chains), improving activity toward VEGFRs and CDK family kinases by engaging deeper hydrophobic pockets. Antifolate analogs rely on N1/N3 nitrogens, the 4‐oxo group, and variations of the glutamate tail (benzoyl, aliphatic, sulfur‐linked) to bind TS, DHFR, and enzymes involved in purine biosynthesis, with tail modifications influencing transport *via* folate carriers and enzyme selectivity. Dual JAK/HDAC inhibitors employ hydroxamic acid groups with suitable pyrazolyl‐alkyl linkers to interact simultaneously with HDAC catalytic zinc ions and JAK hinge residues.

Flexible hydrophobic chains (e.g., piperazine‐linked aromatics) and polar side chains (morpholine, piperazine, sulfonamides) not only enhance solubility and drug‐likeness but also improve interactions with membrane transport proteins, including the multidrug resistance‐associated protein ABCC1, a key player in drug efflux and multidrug resistance. Halogenation on peripheral aryl rings further increases binding affinity via halogen bonding, modulation of electronic density, and π–π stacking within hydrophobic pockets. The choice of linker length, rigidity, and geometry is crucial: short, rigid linkers favor binding to structurally adjacent pockets, whereas longer, flexible linkers allow distinct pharmacophores to engage separate targets. However, excessive flexibility can reduce potency due to entropic penalties. Therefore, achieving a balance between flexibility and preorganization is essential for optimizing multi‐target engagement and overall efficacy.

**Figure 39:**
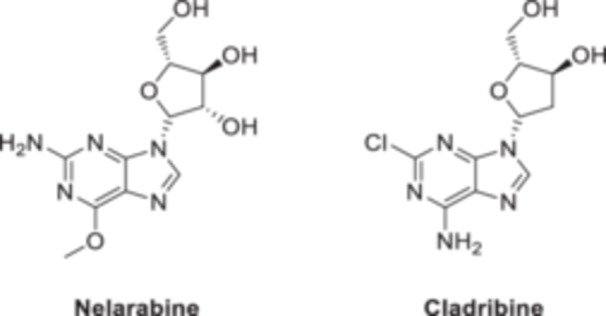
Nelarabine and cladribine structures.

Despite these promising advances, significant challenges remain. Potent enzyme inhibition does not consistently translate into cellular or in vivo activity, often due to high intracellular ATP concentrations, low membrane permeability, or poor pharmacokinetic properties. Moreover, dual‐target optimization can produce conflicting SAR requirements, where modifications that increase potency for one target decrease activity on the other. Some compounds may lack selectivity, raising concerns about potential off‐target effects. Furthermore, linker flexibility may increase conformational entropy and reduce binding affinity, particularly when the interacting pockets differ significantly in spatial orientation or physicochemical environment. This observation underscores the importance of developing dual ligands based on integrated pharmacophores rather than on two scaffolds joined by long, flexible linkers. Future efforts should integrate SAR data with molecular dynamics simulations, structural biology, and predictive ADME profiling to improve selectivity, pharmacokinetics, and translational potential. Importantly, several lead compounds described in this review (e.g., compounds **3**, **8**, **14**, **35**, **54**, and **63r**) demonstrate not only biochemical potency but also significant antiproliferative activity, inducing cell cycle arrest and apoptosis, and, in some cases, in vivo efficacy. The clinical success of approved drugs based on purine and pyrrolo[2,3‐*d*]pyrimidine scaffolds, such as nelarabine, cladribine [83] (Figure 39) and ruxolitinib [48] underscores the therapeutic relevance and druggability of these scaffolds.

In summary, purine and pyrrolo[2,3‐*d*]pyrimidine represent promising and adaptable chemical scaffolds for the development of multitarget‐directed ligands. The capacity to integrate multiple pharmacophores within a single molecule, coupled with well‐characterized SAR principles, enables precise control over target profiles and biological responses. By combining medicinal chemistry, structural biology, and computational modeling, future research can capitalize on these scaffolds to develop next‐generation agents capable of simultaneously modulating complementary or convergent disease pathways. Such compounds could provide effective, low‐toxicity treatments for cancers and other multifactorial diseases where mono‐target therapies have reached their limits.

## Conflicts of Interest

The authors declare no conflicts of interest.

## Data Availability

This article is a review and does not contain any original research data. All data supporting the findings of this study are from previously published sources, which are cited in the manuscript.
